# Feasibility and Sensitivity of Wearable Sensors for Daily Activity Monitoring in Spinal Cord Injury Trials

**DOI:** 10.1177/15459683251352556

**Published:** 2025-07-10

**Authors:** Melina Giagiozis, Irina Lerch, Anita D. Linke, Catherine R. Jutzeler, Rüdiger Rupp, Rainer Abel, Jesús Benito-Penalva, Josina Waldmann, Doris Maier, Michael Baumberger, Jiri Kriz, Andreas Badke, Margret Hund-Georgiadis, Norbert Weidner, László Demkó, Armin Curt

**Affiliations:** 1 Spinal Cord Injury Center, University Hospital Balgrist, Zurich, Switzerland; 2 Department of Health Sciences and Technology (D-HEST), ETH Zurich, Zurich, Switzerland; 3 Swiss Institute of Bioinformatics (SIB), Lausanne, Switzerland; 4 Spinal Cord Injury Center, Heidelberg University Hospital, Heidelberg, Germany; 5 Clinic for Paraplegia, Klinikum Bayreuth GmbH, Bayreuth, Germany; 6 Institut Guttmann, Institut Universitari de Neurorehabilitació adscrit a la UAB, Badalona, Barcelona, Spain; 7 Orthopädische Klinik, Hessisch Lichtenau, Germany; 8 Spinal Cord Injuries, Berufsgenossenschaftliche Unfallklinik Murnau, Murnau, Germany; 9 Swiss Paraplegic Centre, Nottwil, Switzerland; 10 Spinal Cord Unit, Department of Rehabilitation and Sports Medicine, 2nd Faculty of Medicine, Charles University and University Hospital Motol, Prague, Czech Republic; 11 BG Clinic Tübingen, Tübingen, Germany; 12 Clinic of Neurorehabilitation and Paraplegiology, REHAB Basel, Basel, Switzerland

**Keywords:** spinal cord injury, daily activity monitoring, energy expenditure, inertial measurement units, IMUs

## Abstract

**Background:**

The aim of clinical trials for spinal cord injury (SCI) is to improve everyday-life activity outcomes, which requires reliable methods for monitoring patient activity. This study evaluates sensor-derived activity metrics in comparison to established clinical assessment methods.

**Methods:**

Wearable inertial sensors collected data from 69 individuals with acute, traumatic cervical SCI participating in the Nogo-A Inhibition in Spinal Cord Injury trial (NCT03935321), a phase 2b, multicenter, randomized, placebo-controlled trial. During inpatient rehabilitation, participants wore up to 5 inertial sensors for up to 3 consecutive days each week. An estimation of average daily energy expenditure (EE) was used as an indicator of physical activity and compared to the recovery of Upper Extremity Motor Scores (UEMS) and Spinal Cord Independence Measures (SCIM).

**Results:**

Participants in the *verum* (n = 41; 59.4%) and placebo (n = 28; 40.6%) groups showed similar initial activity levels, however, the *verum* group exhibited a significantly greater weekly increase in average daily EE (ΔEE = 11.6 kcal/day/week, 95% CI [1.5, 21.8], *P* = .025). In contrast, no significant group differences were observed in changes in UEMS (ΔUEMS = 0.1/week, 95% CI [−0.2, 0.3], *P* = .603) or SCIM (ΔSCIM = 0.2, per week 95% CI [−0.7, 1.1], *P* = .644).

**Conclusion:**

Continuous sensor-based activity monitoring offers objective and sensitive insights into changes in physical capabilities, effectively complementing periodic clinical assessments. Thus, sensor-derived outcome measures offer potential for improving the evaluation of clinical studies in individuals with SCI.

**Clinical Trail Registration:**

https://clinicaltrials.gov; NCT03935321.

## Introduction

Spinal cord injuries (SCI) profoundly impact motor, sensory, and autonomic functions, leading to significant impairments in mobility, organ regulation, and overall independence.^1,2^ These impairments affect an individual’s ability to perform activities of daily living,^3^ highlighting the need for effective rehabilitation strategies and precise methods to assess recovery. Accurate monitoring of daily physical activity is essential for evaluating functional recovery in individuals with SCI, particularly in the context of clinical trials investigating novel therapeutic interventions.^4^ These trials typically rely on outcome measures such as the International Standards for Neurological Classification of Spinal Cord Injury^5^ and the Spinal Cord Independence Measure (SCIM),^6^ which assess motor and sensory function, and independence in daily activities, respectively. However, these established assessment tools have limitations, including subjectivity, variability between raters, and an inability to capture real-world activity patterns. Consequently, there is a critical need for objective, continuous monitoring tools that can provide reliable insights into recovery and potential treatment effects.

While many activity trackers are commercially available, most are not designed for reliable assessments of individuals with neurological impairments.^7,8^ Therefore, these devices often lack the sensitivity to accurately detect movements such as wheeling or transfers, which are common in individuals with neurological injury, leading to inaccuracies in activity measurements.^9,10^ Time-synchronized inertial measurement units (IMU) offer a promising solution by providing accurate and precise measurements.^11^ These wearable sensors can be placed anywhere on the body, allowing for continuous and objective monitoring of physical activity beyond the confines of clinical settings, such as therapy sessions or movement laboratories.^12^ Previous studies have shown that IMUs can detect complex movement patterns in individuals with SCI^13^ and provide accurate energy expenditure estimates.^14,15^ Moreover, they have demonstrated reliability over multiple days of continuous use, offering reproducible and sensitive measures of physical activity^11^ and functional independence.^12^ Consequently, IMUs can provide valuable insights beyond established clinical assessments.^16^

This study explores the novel application of wearable sensors in clinical trials to provide objective, continuous measures of physical activity in individuals with SCI. By comparing their sensitivity to that of established clinical assessment methods, we aim to determine the added value of inertial sensors for accurately monitoring recovery and evaluating treatment outcomes in a clinical setting. Specifically, we propose 3 primary aims based on data prospectively collected during the Nogo-A Inhibition in Spinal Cord Injury (NISCI) trial, a phase 2b, multicenter, randomized, placebo-controlled trial:

(1) Evaluate the feasibility of sensor-derived measures for capturing activity levels during inpatient rehabilitation for acute, traumatic cervical SCI.
(2) Investigate the relationship between sensor-derived measures and established clinical outcomes.
(3) Assess the sensitivity of sensor-derived metrics in evaluating treatment outcomes in clinical trials.

To achieve these aims, we applied advanced signal processing and machine learning techniques to extract and analyze relevant sensor parameters. By leveraging wearable technology, this study aims to establish IMUs as a feasible tool for accurate and reliable activity tracking in SCI research and rehabilitation.

## Methods

### Study Design and Data Source

Sensor data was collected as part of the NISCI trial (NCT03935321). This was a phase 2b, randomized, placebo-controlled trial conducted at 13 SCI rehabilitation centers in the Czech Republic, Germany, Spain, and Switzerland, with a total enrollment of 126 participants.^17^ The objective of the trial was to analyze efficacy of 6 intrathecal bolus injections of the anti-Nogo-A antibody (NG101)^18^ in individuals with acute, traumatic cervical SCI. The primary outcome was the change in Upper Extremity Motor Score (UEMS)^5^ at 6 months, while a secondary outcome was the change in Spinal Cord Independence Measure (SCIM)^6^ at 6 months. Participants were randomly assigned 2:1 to intrathecal treatment with 45 mg NG101 or placebo (phosphate-buffered saline).^17^

**Figure 1.**
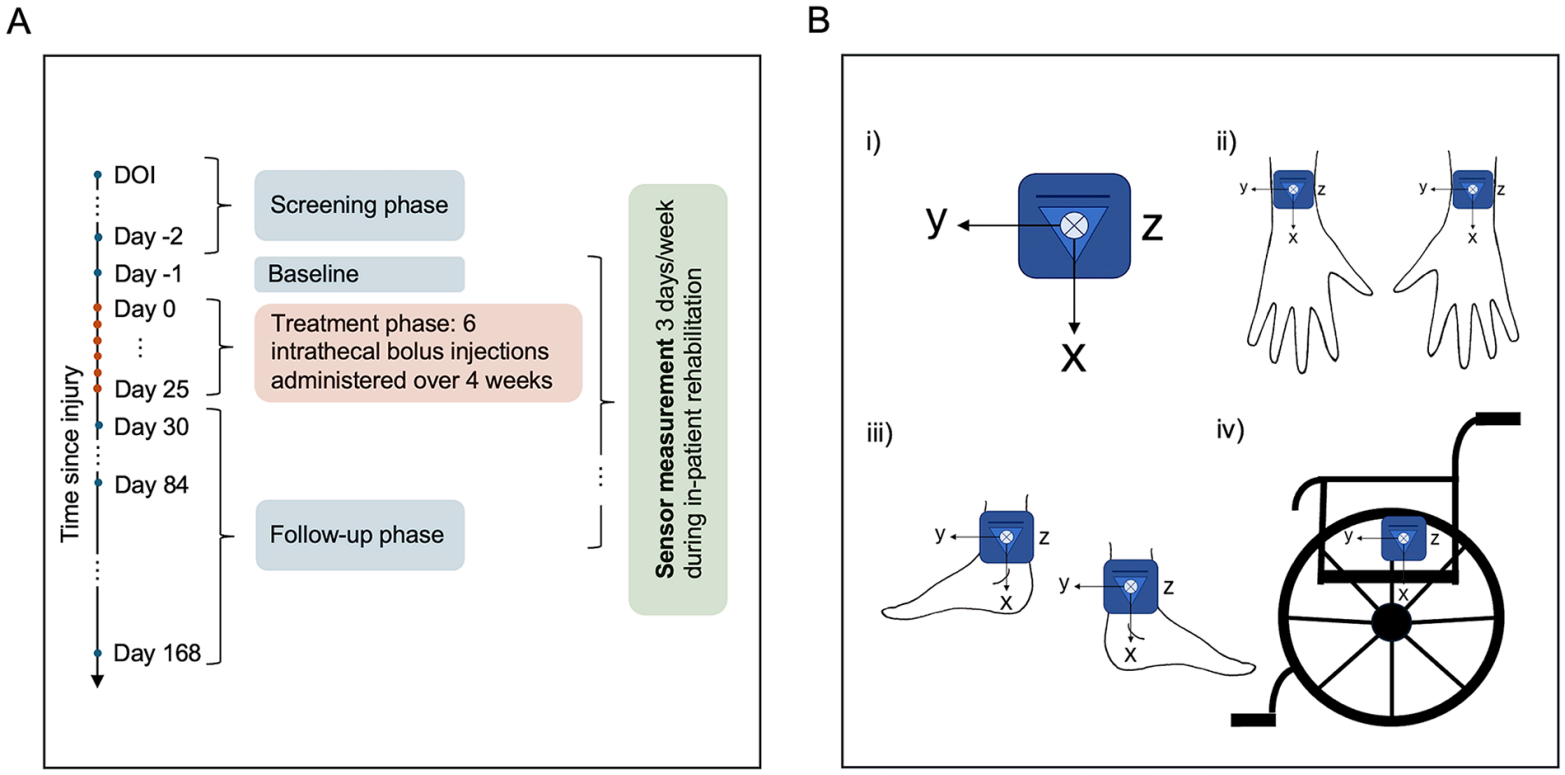
(A) Summary of the study design. Clinical assessments were performed during the screening phase (2-28 days after spinal cord injury), at baseline (the day before treatment initiation), and during the follow-up phase on days 30, 84, and 168. Sensor data was collected during in-patient rehabilitation, with the earliest recordings starting at baseline and the latest extending until discharge from rehabilitation. (B) 5-sensor setup with the local coordinates of each module. (i) IMU modules were placed on (ii) the left/right dorsal wrist, (iii) left/right lateral ankle, and (iv) the wheel of the wheelchair. Abbreviations, DOI, date of injury.

During inpatient rehabilitation, participants were instructed to wear inertial sensors (ZurichMOVE, Switzerland) for up to 3 consecutive days each week.^12^ Data collection could begin as early as baseline (Figure 1(A)), following discharge from intensive care, and continued until their discharge from inpatient rehabilitation. Each participant with a motor incomplete SCI wore 5 modules: 1 attached to the wheelchair wheel, and 1 on each wrist and ankle using flexible straps (Figure 1(B)). For participants with motor complete SCI, the ankle sensors were omitted. Injury completeness was defined according to the American Spinal Injury Association Impairment Scale (AIS).^5^ with AIS A and B classified as motor complete and AIS C and D as motor incomplete. This setup effectively captures movements of the upper and lower extremities, along with data on wheelchair use, providing a comprehensive overview of the participants’ mobility and activity. The IMU modules were time-synchronized via Bluetooth and included a tri-axial accelerometer and a tri-axial gyroscope. To optimize battery life, data was recorded at a reduced sampling frequency of 50 Hz.

### Cohort Definition: Inclusion and Exclusion Criteria

Main inclusion criteria were acute, traumatic SCI within 2 to 28 days before treatment initiation, age 18 to 70 years and a neurological level of injury (NLI) from C1 to C8.^17^ Participants also needed a confirmed classification of AIS A to D^5^ at screening and a UEMS of less than 28 out of 50 at screening to avoid ceiling, according to the unbiased recursive partitioning prediction model.^19^ To be included in our analysis, participants additionally needed to have wearable sensor data collected during the trial period. All other inclusion and exclusion criteria can be found in the trial publication.^17^

### Statistical Analyses

The statistical analysis was performed using Python (version 3.9, MacOS).

An estimate of average daily energy expenditure (EE) was calculated for each multi-day measurement as a proxy for activity levels using IMU-based algorithms, previously developed and validated specifically for individuals with SCI.^14,15^ Resting EE was estimated using the updated Harris-Benedict equation,^20^ while active EE was determined using different models based on motor completeness. For participants with motor complete SCI (AIS A/B), active EE was estimated with 3 sensors (wrists and wheelchair) using a model trained on 24 distinct activities. These activities were classified into 3 categories: low-intensity activities (eg, watching TV, reading, and passive wheeling), high-intensity activities (eg, washing dishes, hand bike ergometer, and weightlifting), and active wheeling-related activities. The classification was performed with a *k*-nearest neighbors algorithm. Following classification, an artificial neural network trained for each activity category was used to estimate active EE.^14^ For individuals with motor incomplete SCI (AIS C/D), active EE was estimated with 5 sensors (wrists, ankles, and wheelchair) using a model trained on 34 distinct activities. A *k*-nearest neighbors algorithm was used to classify activities into 4 different categories, namely “sedentary,” “low-intensity,” “high-intensity,” and “walking.” Following classification, a multiple linear regression model was applied to estimate active EE for each activity class.^15^ Both models were trained and validated using data collected from individuals with SCI, with EE estimated from wearable sensors and validated against gold-standard indirect calorimetry measurements using the Oxycon Mobile system.^14,15^

Five different intensity levels of physical activity were defined based on the metabolic equivalent of task (MET) adapted for individuals with SCI.^21^ REST indicates a MET value of below 1, sedentary activity (SED) corresponds to a MET value between 1 and 1.5, low physical activity (LPA) between 1.5 and 3, moderate physical activity (MPA) from 3 to 6, and vigorous physical activity (VPA) to MET values above 6.^11^ REST, SED, LPA, MPA, and VPA are expressed in percentage of the day spent in each intensity level. A linear regression model was employed to evaluate the change in activity intensity over time across all participants.

The relationship between EE and recovery was evaluated using Spearman’s correlation, determining the strength and direction of associations between average daily EE and conventional clinical scores. Correlations were assessed for the Upper and Lower Extremity Motor Score (UEMS and LEMS, respectively),^5^ the Spinal Cord Independence Measure (SCIM) III,^6^ the Graded Redefined Assessment of Strength, Sensibility, and Prehension V2 (GRASSP),^22^ the Walking Index for Spinal Cord Injury (WISCI) II,^23,24^ the 6-Minute Walk Test (6MWT),^24^ and the sensory assessments Total Pin Prick (TPP) and Total Light Touch (TLT).^5^ Correlation coefficients were interpreted as strong (*r* > .5), moderate (0.3 < *r* ≤ .5), or weak (*r* < .3).^25^ Although sensor data was collected each week, established clinical assessments were conducted periodically at pre-scheduled intervals. To calculate correlations, each recorded average daily EE was matched with the most recent corresponding clinical score, excluding any EE measurements obtained prior to the first clinical assessment. These selected scores, which include both primary and secondary outcome measures of the NISCI trial,^17^ provide a comprehensive clinical overview of an individual’s sensory and motor functions following SCI.

To evaluate how well estimated EE represents an individual’s functional abilities in daily life, we analyzed the relationship between EE and measures of functional independence. Participants were divided into 4 distinct levels of independence (Supplemental Appendix Table A) based on the self-care subscale of the SCIM^6^ at 6 months post-injury.^17^ Level 1 represents severe impairment, while level 4 signifies near-full independence. These levels of independence were evaluated against both the average daily EE at 6 months post-injury and ΔEE. ΔEE reflects the difference in the monthly average of daily EE between the first and sixth months after injury. To address missing EE data at later time points, the last observation carried forward method was used. This is a common approach in longitudinal studies to handle missing data, helping to preserve statistical power and minimize bias.^26^ In fact, it has proven to be one of the most reliable methods of imputation for repeated measures missing after 6 months following SCI.^27^ Statistical comparisons were conducted using the Kruskal–Wallis test (α = .05) with Bonferroni correction for multiple testing.

Lastly, we stratified EE by treatment groups to explore differences in activity and recovery between the NG101 treatment (hereafter referred to as *verum*) and placebo groups. A linear mixed-effects model was applied to analyze the interaction effect between the groups over time, adjusting for age, sex, NLI, study site, first measured EE, time of the first measurement, and total number of measurements. Random effects for each participant and weeks since injury were included to account for both individual differences and changes over time. The treatment groups were further stratified to compare individuals with motor complete (AIS A/B) and incomplete SCI (AIS C/D). To contextualize our findings, we compare the EE results to changes in the UEMS and SCIM, the primary and a secondary outcome of the NISCI trial, respectively.^17^

### Ethics Approval

The investigator-initiated, multicenter, multinational, phase 2b study adhered to Good Clinical Practice guidelines and received approval from the respective ethics committees and institutional review boards. Conducted in accordance with the Declaration of Helsinki, the study followed the ethical guidelines of all participating countries, with policy updates implemented as needed. Written informed consent was obtained from all participants prior to their inclusion in the trial. Activity tracking was included as part of the ethics protocol to monitor participant progress. The study was registered at ClinicalTrials.gov, NCT03935321.

### Data and Code Availability Statement

Anonymized data used in this study will be made available upon request to the corresponding author and in compliance with the General Data Protection Regulation (EU GDPR). The code describing the analysis can be accessed on our GitLab repository: https://gitlab.ethz.ch/BMDSlab/publications/sci/activity-tracking-in-sci-clinical-trials.git.

### Role of Funding Source

The funding sources of this study had no role in study design, data collection, data analysis, data interpretation, or writing of the manuscript. The authors had full access to all the data in the study and had final responsibility for the decision to submit for publication.

## Results

### Subjects

Sensor data was collected from 69 of the 126 subjects with acute, traumatic cervical SCI included in the NISCI trial^17^ during their inpatient rehabilitation or up to 30 weeks after injury (Table 1; Supplemental Appendix Figure A). These participants were treated in 10 of 14 NISCI centers, while the remaining centers did not implement activity trackers due to technical challenges, delays in implementation, and internal regulations. Statistical comparisons between the full cohort and the subset with sensor data revealed no significant differences in age, sex, treatment group distribution, or injury characteristics (Supplemental Appendix Table B).

**Table 1.** Characteristics of the Study Cohort.

Characteristics	Subjects
Number, n	69
Age [years], mean (SD)	49.7 (15.1)
Sex [female], n (%)	11 (15.9)
Treatment group, n (%)	41 (59.4) *verum*
	28 (40.6) placebo
AIS, n (%)	20 (29.0) A
	8 (11.6) B
	25 (36.2) C
	16 (23.2) D
Motor-complete injury, n (%)	28 (40.6)
NLI, n (%)	2 (3.0) C1
	13 (18.8) C2
	15 (21.7) C3
	26 (37.7) C4
	7 (10.1) C5
	6 (8.7) C6
	0 (0) C7
	0 (0) C8
UEMS, mean (SD)	15.2 (7.8)
LEMS, mean (SD)	15.5 (17.4)
SCIM Self-Care, mean (SD)	1.2 (2.6)
SCIM Mobility, mean (SD)	2.3 (6.3)
Participating sites, n (%)	2 (2.9) Barcelona
	5 (7.2) Basel
	25 (36.2) Bayreuth
	4 (5.8) Heidelberg
	4 (5.8) Hessisch-Lichtenau
	4 (5.8) Murnau
	13 (18.8) Nottwil
	2 (2.9) Prague
	2 (2.9) Tübingen
	8 (11.6) Zurich

Abbreviations, SD, standard deviation; AIS, ASIA Impairment Scale; NLI, Neurological Level of Injury; UEMS, Upper Extremity Motor Score; LEMS, Lower Extremity Motor Score; SCIM, Spinal Cord Independence Measure III.

Clinical scores were measured at screening (14.5 ± 6.8 days after injury).

### Average Daily EE During Inpatient Rehabilitation

**Figure 2.**
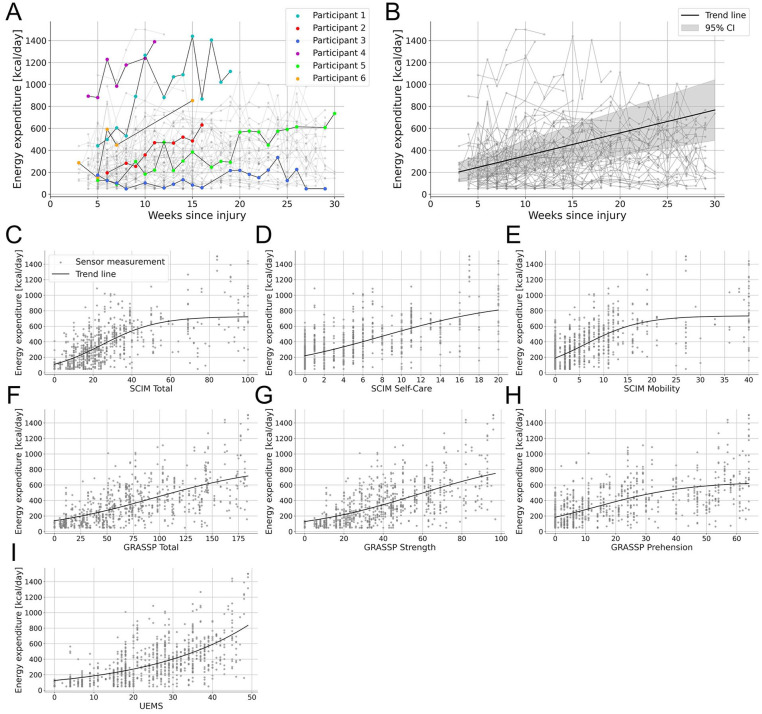
(A) Six representative participants highlighted to show variability in data collection and EE patterns over time. Participant 1 showed high but fluctuating EE with variations up to 500 kcal, while participant 2 had lower EE with a steady increase after injury. The duration of rehabilitation influenced data availability. Participant 3 had sensor data up to 29 weeks, while participant 4, who showed high early activity, was discharged by week 11. Data completeness also varied. Participant 5 had regular weekly measurements with few gaps, whereas participant 6 had large missing segments. (B) Average daily EE of all participants up to 30 weeks post-injury. The trend line illustrates the overall average derived from individual patient trends given by the linear regression model. (C-I) Relationship between the average daily EE and clinical scores with strong correlations (*r* > .5). A logistic model was used to fit the data. Abbreviations, EE, energy expenditure; SCIM, Spinal Cord Independence Measure III; GRASSP, Graded Redefined Assessment of Strength, Sensibility, and Prehension V2; UEMS, Upper Extremity Motor Score.

The analysis included only data collected up to 30 weeks post-injury, which was the maximum duration of enrollment in the NISCI trial.^17^ On average, the observation period between the first and last multi-day measurement was 14.5 ± 8.2 weeks (*verum*: 13.9 ± 8.2, placebo: 15.3 ± 8.2 weeks; Supplemental Appendix Figure B). This resulted in a total of 822 three-day measurements from 69 participants, with an average of 11.9 ± 6.9 measurements per participant. The corresponding coefficient of variation was 58.0%, indicating considerable variability in the number of measurements among participants. Figure 2(A) illustrates the progress of 6 participants, highlighting the individual variability over time (Supplemental Appendix Table C).

For each week of sensor measurements, we calculated the estimated average daily EE. Figure 2B shows an increase in EE over the first 30 weeks after injury, with an average rise of 18.7 kcal/day each week across all participants (Linear Regression Model: *t*(65) = 7.7, slope = 18.7 kcal/day/week, 95% CI [13.9, 23.4] kcal/day/week, *P* < .001; Supplemental Appendix Model A). Furthermore, the first recorded average daily EE was found to be a strong predictor for the final average daily EE measurement (Linear Regression Model: *t*(67) = 10.1, slope = 0.8, 95% CI [0.7, 1.0], *P* < .001; Supplemental Appendix Figure C, Model B). While the overall EE increased over time, activity patterns showed varying trends. Specifically, there was no significant change in the daily percentage of time spent at rest (REST: *t*(65) = −1.4, slope = −0.5% per week, 95% CI [−1.2, 0.2], *P* = .158), in sedentary activity (SED: *t*(65) = −0.7, slope = −0.2%, 95% CI [−0.8, 0.4], *P* = .463), or in vigorous physical activity (VPA: *t*(65) = 1.7, slope = +0.01%, 95% CI [−0.0, 0.0], *P* = .083). In contrast, time spent in light physical activity (LPA: *t*(65) = 4.6, slope = +0.5%, 95% CI [0.3, 0.7], *P* < .001) and moderate physical activity (MPA: *t*(65) = 3.9, slope = +0.2%, 95% CI [0.1, 0.3], *P* < .001) increased significantly (Supplemental Appendix Figure D, Model C).

### Relationship Between EE and Clinical Scores

To investigate the relationship between activity levels and conventional clinical assessments in the first 30 weeks after SCI, Spearman’s correlation between EE and 7 clinical scores was computed (Table 2). Strong correlations were observed between EE and the SCIM^6^ (*r*(816) = .69, *P* < .001), the GRASSP^22^ (*r*(816) = .68, *P* < .001), and the UEMS^5^ (*r*(816) = .63, *P* < .001).^25^ These relationships are visualized in Figure 2(C) to (I), using a logistic model to fit the data. Moderate yet significant correlations were observed between EE and the WISCI^23^ (*r*(816) = .42, *P* < .001), the 6MWT^24^ (*r*(816) = .39, *P* < .001), and the LEMS (*r*(816) = 0.33, *P* < .001).^25^ Similarly, moderate yet significant correlations were noted between EE and TPP (*r*(816) = .37, *P* < .001) and TLT (*r*(816) = .37, *P* < .001).^25^

**Table 2.** Correlations Between EE and Clinical Scores With the Corresponding *P*-values According to a Spearman Rank Correlation Test (α = .05) with Bonferroni Correction.

Clinical score	Spearman correlation coefficient	*P*-values
SCIM total	**.69**	<.001
SCIM Self-care	**.68**	<.001
SCIM Mobility	**.66**	<.001
GRASSP total	**.68**	<.001
GRASSP Strength	**.66**	<.001
GRASSP Prehension	**.66**	<.001
UEMS	**.63**	<.001
WISCI	.42	<.001
6MWT	.39	<.001
TPP	.37	<.001
TLT	.37	<.001
LEMS	.33	<.001

Abbreviations, EE, energy expenditure; SCIM, Spinal Cord Independence Measure III; GRASSP, Graded Redefined Assessment of Strength, Sensibility, and Prehension V2; UEMS, Upper Extremity Motor Score; WISCI, Walking Index for Spinal Cord Injury II; 6MWT, 6-Minute Walk Test; TPP, Total Pin Prick; TLT, Total Light Touch; LEMS, Lower Extremity Motor Score.

*Note*. Strong correlations (*r* > .5) between the average daily EE and clinical scores are indicated in bold font.

**Figure 3.**
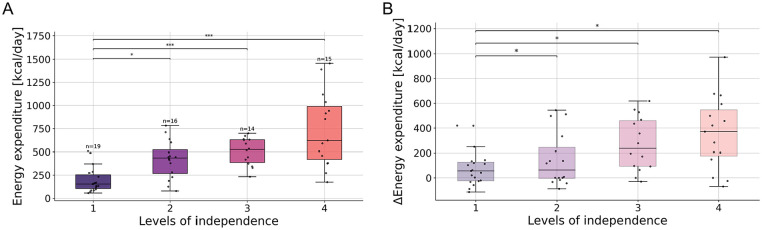
(A) Average daily EE of subjects grouped by level of independence at 6 months after injury. (B) ΔEE of participants grouped by level of independence at 6 months after injury. ΔEE is the difference in average daily EE between the first and sixth month after injury. Abbreviation: EE, energy expenditure. Asterisks represent significant differences with *P < .05; **P < .01; ***P ≤ .00, according to the Kruskal–Wallis test (α = .05) with Bonferroni correction.

To determine whether estimated EE captures real-world upper limb use, participants were divided into 4 distinct levels of independence (Supplemental Appendix Table A) based on the self-care subscale of the SCIM^6^ at 6 months post-injury.^17^ Significant differences in average daily EE at 6 months were observed among the levels of independence (Kruskal–Wallis test: *H*(3) = 28.0, ε² = .445, *P* < .001, Figure 3(A)). Similarly, ΔEE varied significantly across the levels of independence (Kruskal–Wallis test: *H*(3) = 11.9, ε² = .190, *P* = .008, Figure 3(B)).

### Evaluation of Treatment Outcomes Through EE

**Figure 4.**
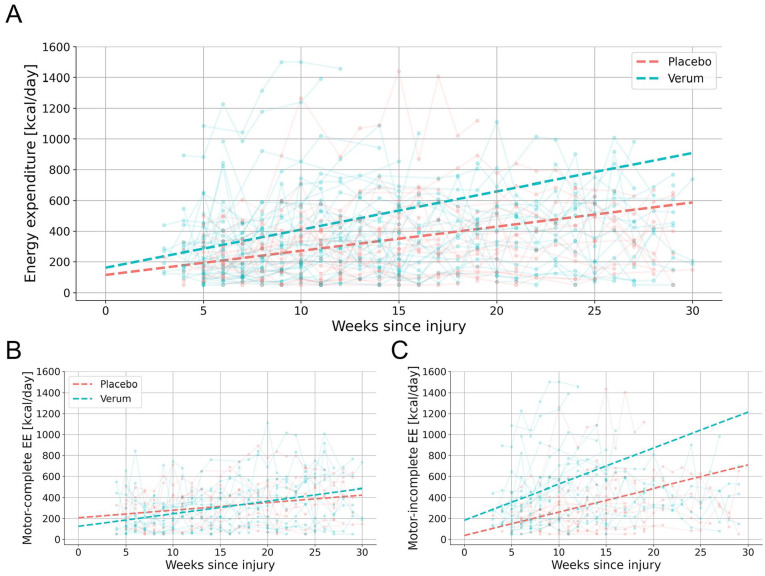
Average daily EE for verum and placebo groups up to 30 weeks post-injury for (A) the entire cohort and stratified by injury type into (B) motor complete and (C) incomplete SCI. The trend lines represent the overall average derived from individual participant trends given by the linear regression model. Abbreviation: EE, energy expenditure.

A linear mixed-effects model was applied to investigate the differences in average daily EE between the treatment groups (*verum* and placebo). A comparison of demographic and clinical data revealed no significant differences between the groups in terms of age, sex, or observation periods. NLI differed but was controlled for in the statistical analysis (Supplemental Appendix Table D). The *verum* group showed a greater increase in EE over time compared to the placebo group (Linear Mixed-Effects Model: ΔEE = 11.6 kcal/day/week, 95% CI [1.5, 21.8], *P* = .025; Supplemental Appendix Model D). On average, the *verum* group exhibited a weekly increase of 11.6 kcal/day more than the placebo group (Figure 4). The UEMS, the primary outcome of the NISCI trial, did not indicate a treatment effect among participants with available sensor measurements (Linear Mixed-Effects Model: ΔUEMS = 0.1, 95% CI [−0.2, 0.3], *P* = .603; Supplemental Appendix Model E). Similarly, the SCIM, a secondary outcome, did not differ between treatment groups (Linear Mixed-Effects Model: ΔSCIM = 0.2, 95% CI [−0.7, 1.1], *P* = .644; Supplemental Appendix Model F).

The treatment groups were further stratified for comparisons between individuals with motor complete (AIS A/B) and incomplete (AIS C/D) SCI (Figure 4). For individuals with motor incomplete SCI, the *verum* group showed a significantly greater increase in EE compared to the placebo group (Linear Mixed-Effects Model: ΔEE = 16.2 kcal/day/week, 95% CI [0.4, 32.1], *P* = .045; Supplemental Appendix Model G). The UEMS (Linear Mixed-Effects Model: ΔUEMS = 0.1, 95% CI [−0.3, 0.5], *P* = .557; Supplemental Appendix Model H) and the SCIM (Linear Mixed-Effects Model: ΔSCIM = 0.01, 95% CI [−1.5, 1.5], *P* = .988; Supplemental Appendix Model I) did not significantly differ between the treatment groups. Among those with motor complete SCI, the *verum* group showed a more moderate but also significantly higher increase of EE (Linear Mixed-Effects Model: ΔEE = 6.6 kcal/day/week, 95% CI [0.2, 13.0], *P* = .044; Supplemental Appendix Model J). However, the UEMS (Linear Mixed-Effects Model: ΔUEMS = 0.01, 95% CI [−0.2, 0.2], *P* = .874; Supplemental Appendix Model K) and the SCIM (Linear Mixed-Effects Model: ΔSCIM = 0.2, 95% CI [−0.3, 0.7], *P* = .450; Supplemental Appendix Model L) did not indicate a treatment effect.

## Discussion

The present study demonstrates the potential of wearable movement sensors to enhance clinical trials by providing continuous, objective data on activity levels. Three main objectives were addressed. Firstly, the feasibility of measuring EE with wearable sensors was established during inpatient rehabilitation for individuals with acute cervical SCI. Secondly, the clinical relevance of sensor-derived data was demonstrated through statistically significant correlations between sensor-based EE estimation and established clinical outcome measures, highlighting the sensitivity of these sensors in revealing functional changes. Finally, the responsiveness of sensor-derived metrics to detect potential treatment effects was assessed. The treatment group experienced a significantly greater increase in average daily EE than the control group in the first 30 weeks post-injury.

### Average Daily EE During Inpatient Rehabilitation

Overall, participants showed an average increase of 19 kcal/day each week during the initial 30 weeks after SCI. However, the rate and pattern of recovery varied depending on the severity of the injury. Clinical factors such as immobilization due to pressure sores, infections, or other complications can limit physical activity and slow recovery.^28^ Additionally, center-specific effects, including differences in rehabilitation programs and intervention intensity, may also play a role in shaping individual recovery trajectories.^29^ Participants increased their time spent in light physical activity (LPA) by 7 minutes/day and moderate physical activity (MPA) by 3 minutes/day each week. Additionally, early activity levels were found to be strong predictors of future activity, indicating that sensor-based metrics can capture early indicators of recovery.

### Relationship Between EE and Clinical Scores

The relationship between average daily EE and clinical scores was evaluated. Findings indicate that activity levels are more strongly associated with motor functions of the upper extremity compared to lower limb and walking abilities. While physical activity in a healthy population typically depends on lower limb function, individuals with SCI primarily rely on upper body strength for activities such as wheeling, transfers, and other daily tasks. Among these, wheeling is particularly demanding, often equaling or surpassing the intensity of moderate to vigorous exercise performed by non-wheelchair users.^30^

A high degree of variability in EE was observed across individuals with similar clinical scores (Figure 2(C)-(I)), likely reflecting differences in daily activity patterns, influenced by personal motivation, rehabilitation intensity, and health-related factors. This highlights the ability of wearable sensors to capture behavioral differences that may not be reflected in standardized clinical assessments. Moreover, individuals with greater recovery showed increased variability in EE, likely due to the broader range of activities available to them. Unlike standardized clinical assessments that may reach a ceiling effect when measuring maximum performance under controlled conditions,^31^ wearable sensors can capture behavioral differences even among individuals with higher levels of recovery.

The impact of functional independence on EE was further evaluated, revealing that 6 months post-injury, individuals with higher activity levels, generally exhibit greater independence. This suggests that sensor-derived activity measures can serve as an indicator of independence, providing insight into how well clinical scores translate into activities of daily living. Moreover, an individual’s level of independence is closely related to their need for caregiving and specialized equipment, and potential for reintegration into society and workforce, all of which carry significant financial implications.^32^ Monitoring activity levels with wearable sensors offers a means to evaluate progress in real-world settings, which can help healthcare providers assess the impact of newly prescribed equipment or adaptations to assistive devices.

### Evaluation of Treatment Outcomes Through EE

To analyze potential treatment effects, activity levels between the *verum* and placebo groups were compared. Individuals in the *verum* group demonstrated a greater increase in activity of 12 kcal/day each week compared to the placebo group, suggesting that the treatment enhanced activity levels beyond natural recovery. This corresponds to a cumulative difference of 360 kcal/day between the *verum* and placebo groups after 30 weeks. Greater physical activity is associated with fewer secondary complications such as pain, fatigue, and depression in individuals with SCI.^33^ Furthermore, accelerated cardiometabolic disease poses a significant health risk in this population.^34^ The increased activity levels after NG101 treatment highlight the drug’s potential in supporting a healthier lifestyle for individuals with SCI.

The treatment groups were further stratified for comparisons between individuals with motor complete and incomplete SCI. Individuals with incomplete injuries in the *verum* group demonstrated a significantly greater increase in activity levels, averaging 16 kcal/day each week, compared to the placebo group. Similarly, individuals with complete injuries in the *verum* group showed a significantly higher increase of 7 kcal/day each week. In the NISCI trial a treatment effect was detected only in participants with incomplete SCI using the UEMS and the SCIM self-care.^17^ In contrast, our analysis revealed a treatment effect based on EE in individuals with both motor incomplete and complete SCI. This suggests that individuals across all injury severities may benefit from NG101 treatment, although the effects appear to be more pronounced in those with motor incomplete SCI.

### Limitations

This study faced several limitations, primarily due to challenges in standardizing multicentric data collection. Differences in data quality and consistency across sites may have affected the reliability and generalizability of our findings. Sensor data was collected from approximately half of the NISCI cohort, with variation in both the number of measurements per participant and the time periods over which they were recorded. The smaller sample size was attributed to technical challenges, delays in implementation, and the inability of some sites to implement sensor-based assessments due to internal regulations. However, the subgroup with available sensor data was demographically and clinically comparable to the full cohort and our sample size remains reasonable for an SCI study.

Furthermore, distinguishing between active and passive movements is often not feasible. Although active and passive wheeling efforts can be differentiated by aligning wheel rotations with hand movements, this does not apply to other activities. During inpatient rehabilitation, individuals with severe impairments receive a considerable amount of passive and assistive therapy, which sensors data cannot distinguish from active movements. Nonetheless, our findings demonstrate that sensor-derived activity metrics offer objective measures of recovery progress.

### Clinical Applications and Future Work

This work is a step toward integrating IMUs in clinical trials to objectively assess the rehabilitation progress of individuals with acute, traumatic SCI, both during inpatient and subsequent outpatient rehabilitation. Wearable sensors offer a non-invasive, cost-effective solution for continuous activity monitoring, with the potential to complement clinical assessments conducted at predefined intervals. Sensor-derived activity data can reveal shifts in recovery progress that may go undetected by established clinical scores. These metrics have the potential to serve as valuable outcome measures or surrogate markers in clinical trials, providing insights into the effects of specific treatments on functional recovery and daily independence.

In future trials, sensor data collected during standardized assessments, such as the 6MWT or a wheeling task, may further help detect subtle changes in functional capacity. While this study focused on real-world activity patterns, adding structured data could improve the sensitivity and interpretability of sensor-based outcomes.

To fully realize the potential of these metrics in clinical trials, however, broader standardization is required. At present, there is no consensus on which sensor-derived metrics best reflect recovery in individuals with SCI. Our study focused on energy expenditure due to its physiological interpretability, and applicability across all levels of injury severity.^14^ However, the clinical relevance of other metrics, such as step count and wheelchair propulsion, should be assessed in future research. In addition to selecting appropriate metrics, determining an optimal monitoring duration is critical. Previous work suggests that 2 days of continuous recording a week are sufficient to obtain reliable estimates in SCI cohorts during inpatient rehabilitation,^11^ although this depends on study design and setting. Moreover, the variability across commercially available IMUs and algorithms poses challenges to cross-study comparability. Establishing standardized protocols will be essential to ensure reproducibility and clinical utility of sensor-derived outcomes in future SCI trials.

## Conclusion

Sensor-derived activity measures can objectively capture individual activity levels beyond the limitations of clinical assessments in individuals with traumatic cervical SCI. Through continuous monitoring, these metrics provide valuable insights into how clinical scores translate into daily functioning in real-world environments. As a complementary assessment, they offer a better understanding of daily activities that established clinical assessments may overlook. Furthermore, integrating sensor-derived metrics into clinical trials can enhance our understanding of recovery progress and treatment outcomes, offering a more comprehensive evaluation.

## Supplemental Material

sj-docx-1-nnr-10.1177_15459683251352556 – Supplemental material for Feasibility and Sensitivity of Wearable Sensors for Daily Activity Monitoring in Spinal Cord Injury Trials Supplemental material, sj-docx-1-nnr-10.1177_15459683251352556 for Feasibility and Sensitivity of Wearable Sensors for Daily Activity Monitoring in Spinal Cord Injury Trials by Melina Giagiozis, Irina Lerch, Anita D. Linke, Catherine R. Jutzeler, Rüdiger Rupp, Rainer Abel, Jesús Benito-Penalva, Josina Waldmann, Doris Maier, Michael Baumberger, Jiri Kriz, Andreas Badke, Margret Hund-Georgiadis, Norbert Weidner, László Demkó and Armin Curt in Neurorehabilitation and Neural Repair

## Abbreviations

AIS American Spinal Injury Association Impairment Scale

EE Energy expenditure

GRASSP Graded Redefined Assessment of Strength, Sensibility, and Prehension

IMU Inertial measurement unit

ISNCSCI International Standards for Neurological Classification of Spinal Cord Injury

LEMS Lower Extremity Motor Score

LPA Low Physical Activity

MET Metabolic Equivalent of Task

MPA Moderate Physical Activity

NG101 Anti-Nogo-A antibody

NISCI Nogo-A Inhibition in Spinal Cord Injury

NLI Neurological level of injury

SCI Spinal cord injury

SCIM Spinal Cord Independence Measure

SED Sedentary activity

TLT Total Light Touch

TPP Total Pin Prick

UEMS Upper Extremity Motor Score

VPA Vigorous Physical Activity

6MWT 6-Minute Walk Test
