# Supplementary material for: Feasibility and Sensitivity of Wearable Sensors for Daily Activity Monitoring in Spinal Cord Injury Trials
Source: Neurorehabil Neural Repair. 2025 Jul 10;39(10):814–25. doi: 10.1177/15459683251352556 (PMC12476463; doi:10.1177/15459683251352556)
Supplement: sj-docx-1-nnr-10.1177_15459683251352556 – Supplemental material for Feasibility and Sensitivity of Wearable Sensors for Daily Activity Monitoring in Spinal Cord Injury Trials [file sj-docx-1-nnr-10.1177_15459683251352556.docx]

##

## **Supplementary Material**

| **Level 1** | 0 – 3 points | Individuals require **continuous personal assistance** and **specialized equipment**, such as motorized wheelchairs and respiratory aids. |
| --- | --- | --- |
| **Level 2** | 4 – 8 points | Individuals have **some independence** but require **substantial support** and adaptations for daily activities. |
| **Level 3** | 9 – 15 points | Individuals demonstrate **improved upper limb function** and can perform limited work, requiring **less frequent assistance**. |
| **Level 4** | 16 – 20 points | Individuals possess **sufficient upper limb function** and **independence** to complete most daily tasks with **minimal or no assistance**. |

## **Table A.** Levels of independence based on the SCIM Self-Care at 6 months after injury. Abbreviations: SCIM: Spinal Cord Independence Measure III.

|  | **Total cohort** | **Sensor cohort** | **p-values** |
| --- | --- | --- | --- |
| Number, n | 126 | 69 |  |
| Age [years], mean (SD) | 46.2 (16.7) | 49.7 (15.1) | 0.207 |
| Sex [female], n (%) | 19 (15.1) | 11 (15.9) | >0.999 |
| Treatment group, n (%) | 78 (61.9) *verum*  48 (38.1) placebo | 41 (59.4) *verum* 28 (40.6) placebo | 0.852 |
| AIS, n (%) | 37 (29.4) A  26 (20.6) B  37 (29.4) C  26 (20.6) D | 20 (29.0) A 8 (11.6) B 25 (36.2) C  16 (23.2) D | 0.409 |
| Motor-complete injury, n (%) | 63 (50.0) | 28 (40.6) | 0.315 |
| NLI, n (%) | 2 (1.6) C1  17 (13.5) C2  26 (20.6) C3  52 (41.3) C4  20 (15.9) C5  9 (7.1) C6  0 (0) C7  0 (0) C8 | 2 (3.0) C1  13 (18.8) C2  15 (21.7) C3  26 (37.7) C4  7 (10.1) C5  6 (8.7) C6  0 (0) C7  0 (0) C8 | 0.766 |
| UEMS, mean (SD) | 14.3 (7.9) | 15.2 (7.8) | 0.695 |
| LEMS, mean (SD) | 12.9 (16.6) | 15.5 (17.4) | 0.292 |
| SCIM Self-Care, mean (SD) | 0.8 (2.1) | 1.2 (2.6) | 0.236 |
| SCIM Mobility, mean (SD) | 1.7 (4.9) | 2.3 (6.3) | 0.305 |
| URP nodes, n (%) | 14 (11.1) Nodes 4–5  9 (7.1) Nodes 8–9  22 (17.5) Node 10  39 (31.0) Node 13  42 (33.3) Nodes 16–18 | 5 (7.2) Nodes 4–5  2 (2.9) Nodes 8–9  15 (21.7) Node 10  21 (30.4) Node 13  26 (37.8) Nodes 16–18 | 0.595 |

## **Table B.** Characteristics of the entire NISCI cohort and the subset of subjects with sensor measurements. Corresponding p-values were calculated using the Mann-Whitney U test for continuous and the Chi-squared test for categorical variables. Clinical scores were measured at screening (total cohort: 15.5 ± 6.8, sensor cohort: 14.5 ± 6.8 days after injury). Abbreviations: AIS: ASIA Impairment Scale; NLI: Neurological Level of Injury; UEMS: Upper Extremity Motor Score; LEMS: Lower Extremity Motor Score; SCIM: Spinal Cord Independence Measure III, URP nodes: Nodes created by the unbiased recursive partitioning prediction model used to stratify the treatment groups.

#

| **Participant** | **AIS, NLI @BL** | **UEMS @BL** | **UEMS @FU** | **SCIM@BL** | **SCIM@FU** |
| --- | --- | --- | --- | --- | --- |
| **Participant 1** | D, C5 | 26 | 50 | 27 | 100 |
| **Participant 2** | C, C4 | 23 | 41 | 12 | 94 |
| **Participant 3** | A, C3 | 3 | 11 | 0 | 15 |
| **Participant 4** | D, C1 | 28 | 49 | 33 | 90 |
| **Participant 5** | A, C4 | 24 | 29 | 14 | 36 |
| **Participant 6** | C, C3 | 16 | 37 | 10 | 96 |

##

## **Table C.** Characteristics of example participants of [Figure 2A](#_fbgn773377m1). Abbreviations: AIS: ASIA Impairment Scale; NLI: Neurological Level of Injury; UEMS: Upper Extremity Motor Score; SCIM: Spinal Cord Independence Measure III, BL: Baseline of the NISCI trial; FU: 6-month Follow-up of the NISCI trial.

##

|  | ***Verum* subjects** | **Placebo subjects** | **p-values** |
| --- | --- | --- | --- |
| Number, n | 41 | 28 |  |
| Age [years], mean (SD) | 47.5 (15.8) | 52.9 (13.2) | 0.195 |
| Sex [female], n (%) | 7 (17.1) | 4 (14.3) | >0.999 |
| AIS, n (%) | 13 (31.7) A 4 (9.8) B 15 (36.6) C  9 (21.9) D | 7 (25.0) A 4 (14.3) B  10 (35.7) C  7 (25.0) D | 0.891 |
| Motor-complete injury, n (%) | 17 (41.5) | 11 (39.3) | 0.891 |
| NLI, n (%) | 2 (4.9) C1  11 (26.8) C2  7 (17.1) C3  16 (39.0) C4  1 (2.4) C5  4 (9.8) C6  0 (0) C7 | 0 (0) C1  2 (7.1) C2  8 (28.6) C3  10 (35.8) C4  6 (21.4) C5  2 (7.1) C6  0 (0) C7 | 0.036 |
| UEMS, mean (SD) | 17.7 (10.1) | 18.5 (8.2) | >0.999 |
| LEMS, mean (SD) | 17.6 (19.3) | 15.8 (16.8) | >0.999 |
| SCIM Self-care, mean (SD) | 6.2 (5.0) | 9.9 (6.1) | >0.999 |
| SCIM Mobility, mean (SD) | 15.9 (10.3) | 14.4 (5.1) | >0.999 |
| URP nodes, n (%) | 4 (9.8) Nodes 4–5  1 (2.4) Nodes 8–9  8 (19.5) Node 10  12 (29.3) Node 13  16 (39.0) Nodes 16–18 | 1 (3.6) Nodes 4–5  1 (3.6) Nodes 8–9  7 (25.0) Node 10  9 (32.1) Node 13  10 Nodes (35.7) 16–18 | 0.865 |
| Sensor data observation period [weeks], mean (SD) | 13.9 (8.2) | 15.3 (8.2) | 0.520 |

##

## **Table D.** Characteristics of the *verum* and placebo cohorts of subjects with sensor measurements. The corresponding p-values were calculated using the Mann-Whitney U test for continuous and the Chi-squared test for categorical variables. Clinical scores were measured at screening (*verum*: 12.6 ± 6.5, placebo: 17.3 ± 6.3 days after injury). Abbreviations: AIS: ASIA Impairment Scale; NLI: Neurological Level of Injury; UEMS: Upper Extremity Motor Score; LEMS: Lower Extremity Motor Score; SCIM: Spinal Cord Independence Measure III, URP nodes: Nodes created by the unbiased recursive partitioning prediction model used to stratify the treatment groups.

## **Figure A.** Flowchart of participant inclusion. Of the 129 participants randomized in the NISCI trial, 69 contributed sensor data (41 in the NG101 group and 28 in the placebo group) and were included in the full analysis set
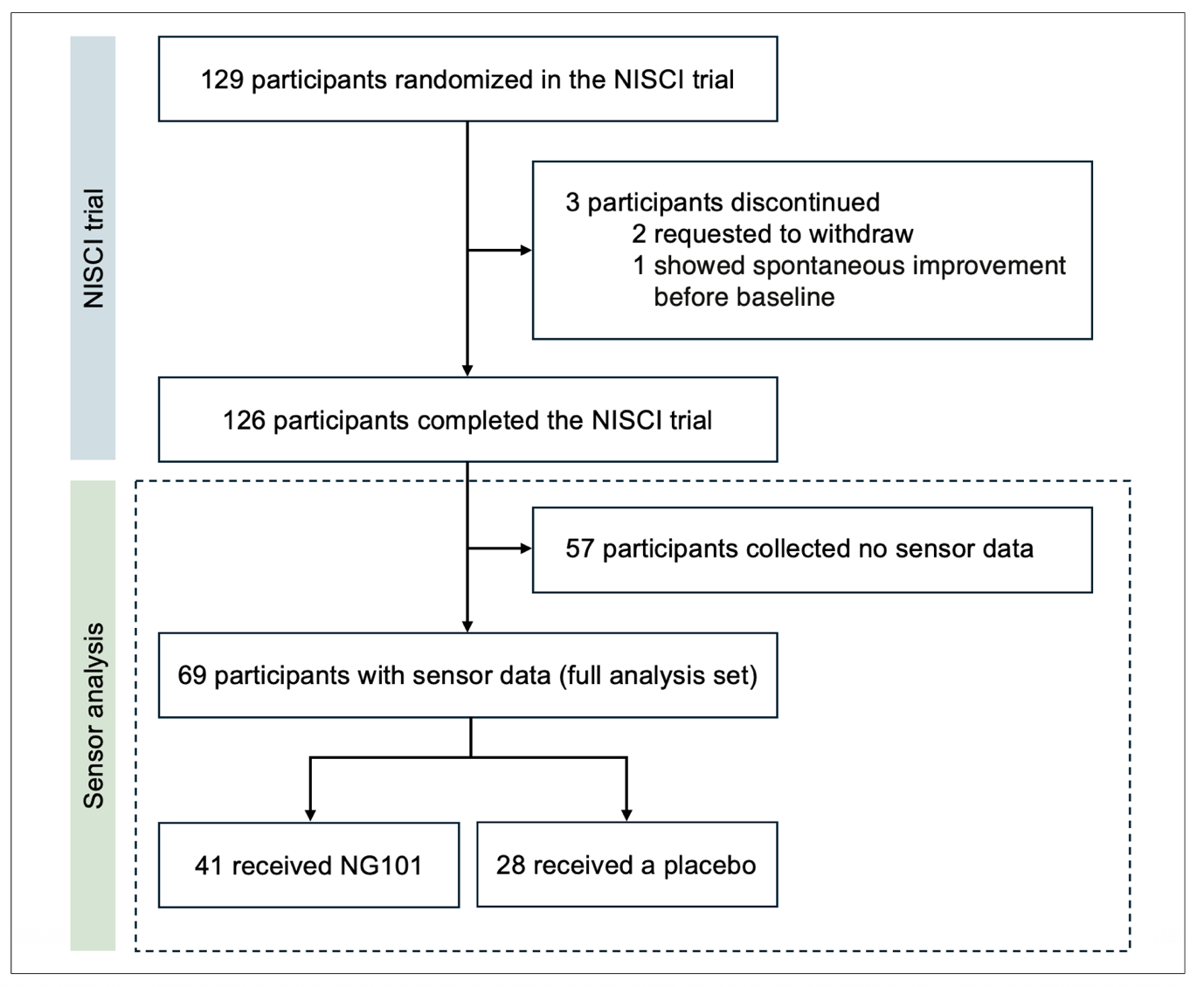
.

##

##

##

## **
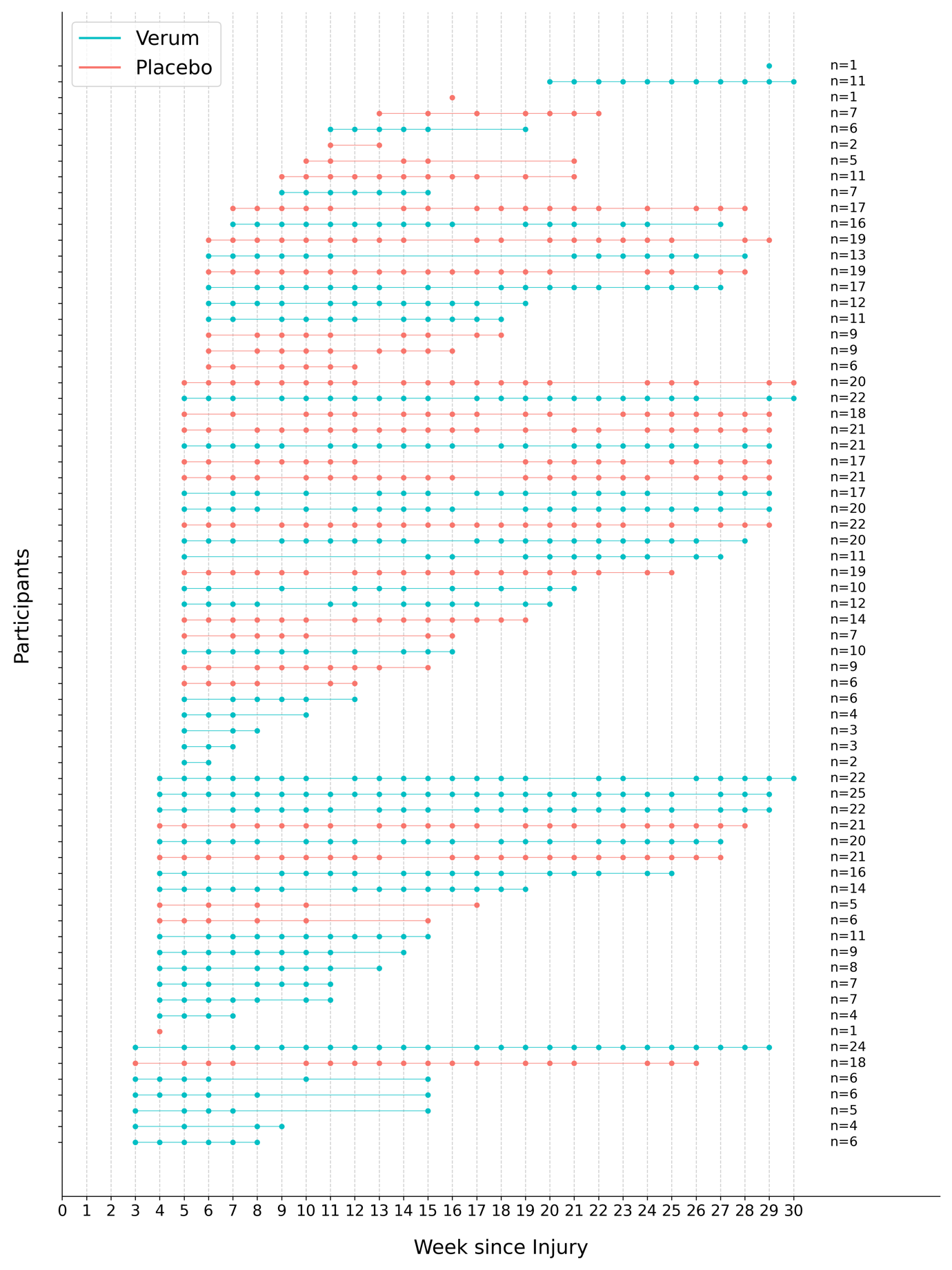
Figure B.** Observation periods for individual participants, distinguished between *verum* and placebo groups. Participants are sorted by the timing of their first recorded measurement and, in cases of ties, by their last recorded measurement. Each marker represents a week in which a multi-day sensor measurement was conducted, with the total number of measurements noted on the right. Abbreviations: EE: energy expenditure.


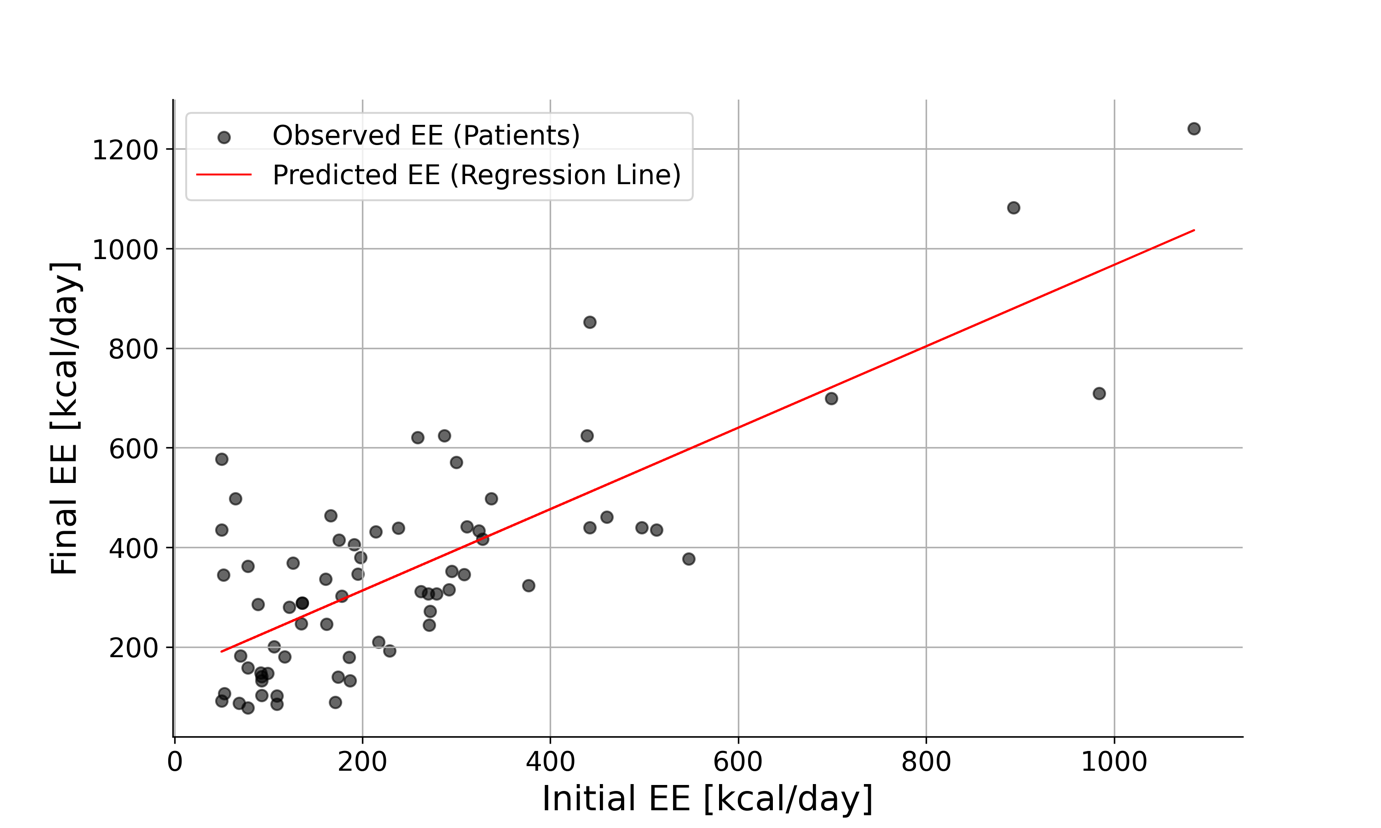


##

## **Figure C.** Predictive relationship between the first and the last average daily EE measurement. The proximity of the observed EE to the regression line reflects the model's fit. Abbreviations: EE: energy expenditure.


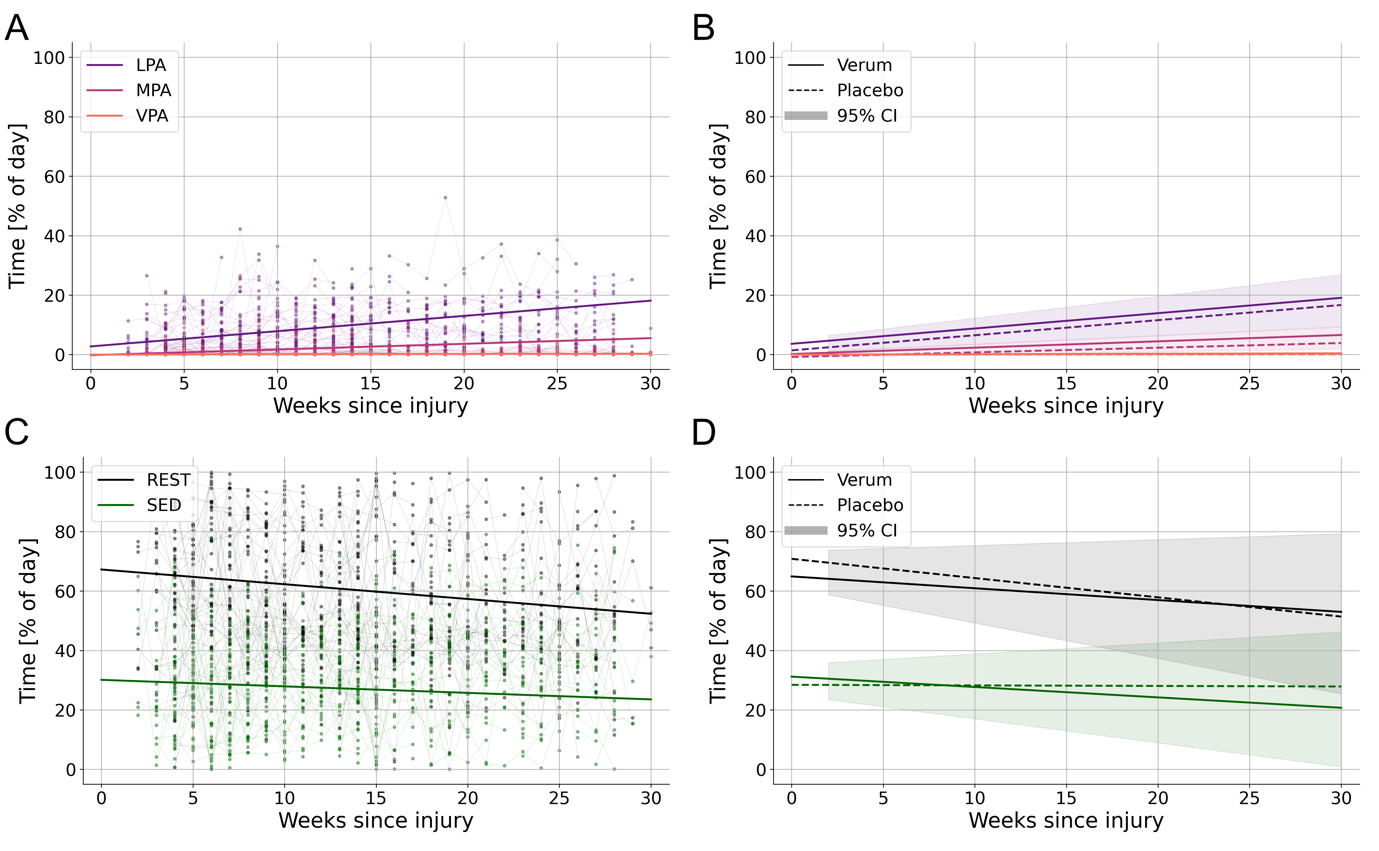


##

## **Figure D.** Average daily percentage of time spent in LPA, MPA, and VPA **(A)** across all participants and **(B)** for the *verum* and placebo groups up to 30 weeks post-injury. Average daily percentage of time spent in SED and at REST **(C)** across all participants and **(D)** for the *verum* and placebo groups up to 30 weeks post-injury. The trend lines represent the overall average derived from individual trends given by the linear regression model. Abbreviations: LPA: low physical activity; MPA: moderate physical activity; VPA: vigorous physical activity; SED: sedentary activity (low-energy activity); REST: resting (inactivity).

| **Participant** | **Slope [kcal/week]** | **Intercept [kcal]** | **95% CI (Slope)** | **p-value** | **n** |
| --- | --- | --- | --- | --- | --- |
| **Participant 1** | 5.8 | 11.3 | [-18.6, 30.3] | 0.640 | 5 |
| **Participant 2** | 31.9 | 0.0 | [-24.9, 88.7] | 0.271 | 7 |
| **Participant 3** | 0.0 | 662.2 | [-98.6, 98.6] | 1.000 | 17 |
| **Participant 4** | 7.7 | 221.0 | [-34.1, 49.5] | 0.717 | 5 |
| **Participant 5** | N/A |  | N/A | N/A | 1 |
| **Participant 6** | 50.9 | 99.0 | [-163.2, 265.1] | 0.641 | 5 |
| **Participant 7** | 23.7 | 406.3 | [-116.5, 164.0] | 0.740 | 6 |
| **Participant 8** | 2.5 | 381.2 | [-48.8, 53.8] | 0.925 | 21 |
| **Participant 9** | 19.1 | 321.2 | [-60.6, 98.8] | 0.639 | 21 |
| **Participant 10** | 13.1 | 299.3 | [-35.3, 61.5] | 0.596 | 14 |
| **Participant 11** | 22.8 | 0.0 | [-56.1, 101.7] | 0.571 | 22 |
| **Participant 12** | 13.9 | 0.0 | [-60.3, 88.0] | 0.714 | 9 |
| **Participant 13** | 36.2 | 0.0 | [-51.7, 124.2] | 0.419 | 9 |
| **Participant 14** | 2.2 | 279.9 | [-30.9, 35.3] | 0.896 | 22 |
| **Participant 15** | 27.6 | 4.8 | [-51.5, 106.7] | 0.494 | 12 |
| **Participant 16** | 0.0 | 315.8 | [-81.1, 81.1] | 1.000 | 6 |
| **Participant 17** | 5.2 | 71.4 | [-28.8, 39.2] | 0.763 | 22 |
| **Participant 18** | 2.9 | 87.6 | [-30.0, 35.8] | 0.862 | 20 |
| **Participant 19** | 1.8 | 168.6 | [-30.2, 33.8] | 0.914 | 18 |
| **Participant 20** | 2.2 | 52.5 | [-13.2, 17.7] | 0.780 | 16 |
| **Participant 21** | 55.9 | 0.0 | [-160.6, 272.3] | 0.613 | 10 |
| **Participant 22** | 0.0 | 407.3 | [-106.7, 106.7] | 1.000 | 3 |
| **Participant 23** | 48.5 | 55.0 | [-63.1, 160.0] | 0.394 | 11 |
| **Participant 24** | 76.5 | 0.0 | [-125.1, 278.1] | 0.457 | 9 |
| **Participant 25** | 59.6 | 0.0 | [-63.1, 182.2] | 0.341 | 6 |
| **Participant 26** | 18.2 | 0.0 | [-57.5, 93.9] | 0.637 | 17 |
| **Participant 27** | 64.9 | 640.3 | [-69.1, 198.9] | 0.342 | 7 |
| **Participant 28** | 70.4 | 717.6 | [-81.3, 222.2] | 0.363 | 6 |
| **Participant 29** | 64.1 | 0.0 | [-16.3, 144.4] | 0.118 | 2 |
| **Participant 30** | 1.0 | 167.7 | [-20.4, 22.4] | 0.927 | 3 |
| **Participant 31** | 52.3 | 92.6 | [-5.5, 110.1] | 0.076 | 4 |
| **Participant 32** | 1.9 | 72.3 | [-12.9, 16.7] | 0.804 | 20 |
| **Participant 33** | 7.8 | 137.5 | [-53.0, 68.6] | 0.801 | 6 |
| **Participant 34** | 9.0 | 69.8 | [-71.7, 89.8] | 0.826 | 16 |
| **Participant 35** | 16.7 | 99.0 | [-50.4, 83.8] | 0.626 | 6 |
| **Participant 36** | N/A |  | N/A | N/A | 1 |
| **Participant 37** | 4.6 | 37.0 | [-22.4, 31.6] | 0.740 | 19 |
| **Participant 38** | 7.4 | 31.8 | [-63.3, 78.2] | 0.837 | 10 |
| **Participant 39** | 0.0 | 470.3 | [-137.1, 137.1] | 1.000 | 6 |
| **Participant 40** | 20.4 | 0.0 | [-176.4, 217.2] | 0.839 | 2 |
| **Participant 41** | N/A |  | N/A | N/A | 1 |
| **Participant 42** | 22.4 | 80.9 | [-114.7, 159.5] | 0.749 | 20 |
| **Participant 43** | 18.6 | 21.0 | [-66.0, 103.2] | 0.666 | 11 |
| **Participant 44** | 13.7 | 215.8 | [-88.7, 116.1] | 0.793 | 8 |
| **Participant 45** | 26.1 | 0.0 | [-109.2, 161.4] | 0.706 | 13 |
| **Participant 46** | 20.4 | 75.7 | [-90.7, 131.5] | 0.719 | 11 |
| **Participant 47** | 8.6 | 209.3 | [-63.3, 80.5] | 0.815 | 21 |
| **Participant 48** | 14.0 | 219.6 | [-84.1, 112.1] | 0.780 | 19 |
| **Participant 49** | 10.7 | 188.7 | [-61.3, 82.7] | 0.771 | 17 |
| **Participant 50** | 7.1 | 420.4 | [-55.8, 70.0] | 0.824 | 19 |
| **Participant 51** | 17.1 | 96.6 | [-60.9, 95.1] | 0.668 | 21 |
| **Participant 52** | 11.2 | 440.4 | [-92.9, 115.3] | 0.833 | 20 |
| **Participant 53** | 23.2 | 195.5 | [-72.1, 118.5] | 0.633 | 12 |
| **Participant 54** | 13.0 | 0.0 | [-35.7, 61.8] | 0.600 | 4 |
| **Participant 55** | 4.8 | 0.0 | [-33.0, 42.5] | 0.805 | 11 |
| **Participant 56** | 0.0 | 422.7 | [-48.5, 48.5] | 1.000 | 6 |
| **Participant 57** | 22.1 | 0.0 | [-68.4, 112.7] | 0.632 | 7 |
| **Participant 58** | 25.7 | 164.7 | [-144.4, 195.8] | 0.767 | 7 |
| **Participant 59** | 13.2 | 320.0 | [-145.4, 171.9] | 0.870 | 4 |
| **Participant 60** | 0.0 | 67.0 | [-24.6, 24.6] | 1.000 | 9 |
| **Participant 61** | 0.0 | 199.9 | [-46.2, 46.2] | 1.000 | 7 |
| **Participant 62** | 10.7 | 105.6 | [-40.1, 61.5] | 0.680 | 24 |
| **Participant 63** | 3.4 | 179.6 | [-29.0, 35.7] | 0.837 | 22 |
| **Participant 64** | 2.2 | 69.4 | [-22.6, 27.0] | 0.861 | 17 |
| **Participant 65** | 1.0 | 296.1 | [-43.9, 45.8] | 0.966 | 21 |
| **Participant 66** | 16.1 | 162.2 | [-54.1, 86.4] | 0.653 | 18 |
| **Participant 67** | 52.5 | 304.6 | [-112.0, 216.9] | 0.532 | 14 |
| **Participant 68** | 7.0 | 191.0 | [-39.2, 53.1] | 0.767 | 25 |
| **Participant 69** | 18.3 | 22.6 | [-78.1, 114.7] | 0.710 | 11 |
| **Overall** | 18.7 | 167.4 | [13.9, 23.4] | **<0.001** | 65 |

## **Model A.** Linear regression analysis results for EE trends in individual participants. Significances (p <0.05) are indicated in bold font. The p-values indicate whether the slope of EE over time is significantly different from zero for each participant. Abbreviations: EE: energy expenditure; CI: confidence interval; n: sample size, i.e. number of multi-day measurements.

| **Metric** | **Slope [kcal/week]** | **Intercept [kcal]** | **95% CI (Slope)** | **p-value** | **df** |
| --- | --- | --- | --- | --- | --- |
| Value | 0.8 | 154.0 | [0.7, 1.0] | **<0.001** | 67 |

## **Model B.** Linear regression model results showing the predictive relationship between the first recorded average daily EE and the final average daily EE measurement. Significances (p <0.05) are indicated in bold font. Abbreviations: EE: energy expenditure; CI: confidence interval; df: degrees of freedom, i.e. number of independent values that can vary in the statistical analysis.

| **Intensity** | **Slope [% of day per week]** | **Intercept [% of day]** | **95% CI (Slope)** | **p-value** | **df** |
| --- | --- | --- | --- | --- | --- |
| REST | -0.5 | 67.2 | [-1.2, 0.2] | 0.158 | 65 |
| SED | -0.2 | 30.1 | [-0.8, 0.4] | 0.463 | 65 |
| LPA | 0.5 | 2.8 | [0.3, 0.7] | **<0.001** | 65 |
| MPA | 0.2 | -0.2 | [0.1, 0.3] | **<0.001** | 65 |
| VPA | 0.01 | -0.02 | [-0.0, 0.0] | 0.083 | 65 |

##

## **Model C.** Linear regression model results showing the estimated weekly changes in time spent at different activity intensities (REST, SED, LPA, MPA, and VPA) over the first 30 weeks after injury. Significances (p <0.05) are indicated in bold font. Abbreviations: REST: rest time (inactive); SED: sedentary time (low-energy activity); LPA: low physical activity; MPA: moderate physical activity; VPA: vigorous physical activity; CI: confidence interval; df: degrees of freedom, i.e. number of independent values that can vary in the statistical analysis.

##

| **Variable** | **Coef.** | **Std. Err.** | **z** | **P>\|z\|** | **[0.025** | **0.975]** | **Effect** |
| --- | --- | --- | --- | --- | --- | --- | --- |
| **Baseline (Intercept)** | 18.7 | 101.1 | 0.185 | 0.853 | -179.4 | 216.8 | Fixed |
| **Treatment Group (*Verum*)** | -50.2 | 35.2 | -1.427 | 0.154 | -119.2 | 18.8 | Fixed |
| **Site: Barcelona** | -35.2 | 100.8 | -0.35 | 0.727 | -232.8 | 162.3 | Fixed |
| **Site: Basel** | -31.5 | 62.2 | -0.507 | 0.612 | -153.4 | 90.4 | Fixed |
| **Site: Heidelberg** | 35.4 | 70.7 | 0.501 | 0.616 | -103.2 | 174.1 | Fixed |
| **Site: Hessisch Lichtenau** | 95.2 | 73.2 | 1.3 | 0.194 | -48.3 | 238.7 | Fixed |
| **Site: Murnau** | 10.8 | 62.2 | 0.174 | 0.862 | -111.1 | 132.8 | Fixed |
| **Site: Nottwil** | 70.1 | 41.7 | 1.684 | 0.092 | -11.5 | 151.8 | Fixed |
| **Site: Prague** | -3.9 | 85.5 | -0.045 | 0.964 | -171.5 | 163.8 | Fixed |
| **Site: Tübingen** | 102.6 | 109.0 | 0.941 | 0.347 | -111.1 | 316.3 | Fixed |
| **Site: Zurich** | 31.4 | 47.0 | 0.668 | 0.504 | -60.7 | 123.4 | Fixed |
| **NLI: C2** | -1.4 | 25.0 | -0.057 | 0.954 | -50.5 | 47.6 | Fixed |
| **NLI: C3** | -2.2 | 24.4 | -0.09 | 0.928 | -50.1 | 45.7 | Fixed |
| **NLI: C4** | -2.2 | 24.9 | -0.089 | 0.929 | -51.1 | 46.7 | Fixed |
| **NLI: C5** | -0.9 | 25.7 | -0.035 | 0.972 | -51.3 | 49.5 | Fixed |
| **NLI: C6** | -1.7 | 26.1 | -0.064 | 0.949 | -52.9 | 49.6 | Fixed |
| **NLI: C7** | -1.1 | 28.3 | -0.04 | 0.968 | -56.6 | 54.3 | Fixed |
| **NLI: no NLI** | -0.4 | 44.3 | -0.01 | 0.992 | -87.3 | 86.5 | Fixed |
| **Week since injury** | 11.7 | 4.0 | 2.937 | **0.003** | 3.9 | 19.5 | Fixed |
| **Interaction Effect:**  **Week x Treatment Group** | 11.6 | 5.2 | 2.246 | **0.025** | 1.5 | 21.8 | Fixed |
| **Age at Injury** | -0.4 | 1.0 | -0.387 | 0.699 | -2.4 | 1.6 | Fixed |
| **Sex (Male=1, Female=0)** | 75.2 | 46.0 | 1.635 | 0.102 | -15.0 | 165.3 | Fixed |
| **Number of Measurements** | 0.3 | 2.7 | 0.103 | 0.918 | -5.0 | 5.5 | Fixed |
| **First measured EE** | 0.8 | 0.1 | 11.206 | **<0.001** | 0.7 | 1.0 | Fixed |
| **Week of first measured EE** | -17.5 | 7.0 | -2.511 | **0.012** | -31.2 | -3.8 | Fixed |
| **Participant-Specific EE Variability** | 0.9 | 0.2 |  |  |  |  | Random |
| **Covariance: Intercept and Week** | -0.1 | 0.0 |  |  |  |  | Random |
| **Week-Specific EE Variability** | 0.0 | 0.0 |  |  |  |  | Random |

##

## **Model D.** Mixed linear model analyzing the relationship between weekly EE and key predictors, including treatment group, clinical site, and demographic factors. Fixed effects include treatment group, site, week since injury, age at injury, sex, NLI, and related interactions. Random effects account for participant-specific variability and changes over time in intercepts and slopes. Bayreuth was selected as the reference site because it had the largest cohort. Significances (p <0.05) are indicated in bold font. Abbreviations: EE: energy expenditure; NLI: Neurological Level of Injury; Coef: coefficient; Std. Err.: standard error.

| **Variable** | **Coef.** | **Std. Err.** | **z** | **P>\|z\|** | **[0.025** | **0.975]** | **Effect** |
| --- | --- | --- | --- | --- | --- | --- | --- |
| **Baseline (Intercept)** | 26.1 | 8.7 | 3.011 | **0.003** | 9.1 | 43.1 | Fixed |
| **Treatment Group (*Verum*)** | -2.4 | 3.0 | -0.802 | 0.423 | -8.4 | 3.5 | Fixed |
| **Site: Barcelona** | 2.0 | 12.8 | 0.153 | 0.878 | -23.1 | 27.0 | Fixed |
| **Site: Basel** | -2.6 | 5.9 | -0.435 | 0.664 | -14.2 | 9.0 | Fixed |
| **Site: Heidelberg** | -3.9 | 7.1 | -0.549 | 0.583 | -17.9 | 10.1 | Fixed |
| **Site: Hessisch Lichtenau** | -2.6 | 6.9 | -0.384 | 0.701 | -16.1 | 10.8 | Fixed |
| **Site: Murnau** | 5.5 | 6.5 | 0.859 | 0.390 | -7.1 | 18.2 | Fixed |
| **Site: Nottwil** | -1.5 | 3.9 | -0.392 | 0.695 | -9.1 | 6.1 | Fixed |
| **Site: Prague** | 4.5 | 8.3 | 0.538 | 0.591 | -11.9 | 20.9 | Fixed |
| **Site: Tübingen** | -4.3 | 9.7 | -0.441 | 0.659 | -23.4 | 14.8 | Fixed |
| **Site: Zurich** | -1.9 | 4.5 | -0.416 | 0.677 | -10.8 | 7.0 | Fixed |
| **NLI: C2** | 0.0 | 0.4 | 0.012 | 0.990 | -0.8 | 0.8 | Fixed |
| **NLI: C3** | 0.0 | 0.4 | 0.013 | 0.990 | -0.8 | 0.8 | Fixed |
| **NLI: C4** | 0.0 | 0.4 | 0.014 | 0.989 | -0.8 | 0.8 | Fixed |
| **NLI: C5** | 0.0 | 0.4 | 0.029 | 0.977 | -0.8 | 0.9 | Fixed |
| **NLI: C6** | 0.0 | 0.4 | 0.03 | 0.976 | -0.8 | 0.9 | Fixed |
| **NLI: C7** | 0.0 | 0.5 | 0.029 | 0.977 | -0.9 | 0.9 | Fixed |
| **NLI: no NLI** | 0.0 | 0.7 | 0.016 | 0.987 | -1.4 | 1.5 | Fixed |
| **Week since injury** | 0.3 | 0.1 | 3.934 | **<0.001** | 0.2 | 0.5 | Fixed |
| **Interaction Effect:**  **Week x Treatment Group** | 0.1 | 0.1 | 0.521 | 0.603 | -0.2 | 0.3 | Fixed |
| **Age at Injury** | -0.1 | 0.1 | -0.812 | 0.417 | -0.3 | 0.1 | Fixed |
| **Sex (Male=1, Female=0)** | 1.3 | 4.4 | 0.303 | 0.762 | -7.2 | 9.9 | Fixed |
| **Number of Measurements** | -0.2 | 0.3 | -0.705 | 0.481 | -0.7 | 0.3 | Fixed |
| **First measured EE** | 0.0 | 0.0 | 3.558 | **<0.001** | 0.0 | 0.0 | Fixed |
| **Week of first measured EE** | -0.7 | 0.7 | -1.075 | 0.282 | -2.1 | 0.6 | Fixed |
| **Participant-Specific EE Variability** | 26.9 | 5.7 |  |  |  |  | Random |
| **Covariance: Intercept and Week** | 0.1 | 0.2 |  |  |  |  | Random |
| **Week-Specific EE Variability** | 0.0 | 0.0 |  |  |  |  | Random |

##

## **Model E.** Mixed linear model analyzing the relationship between UEMS and key predictors for the entire cohort with sensor data. Fixed effects include treatment group, clinical site, week since injury, age at injury, sex, NLI, and related interactions. Random effects account for participant-specific variability and changes over time in intercepts and slopes. Bayreuth was selected as the reference site because it had the largest cohort. Significances (p <0.05) are indicated in bold font. Abbreviations: UEMS: Upper Extremity Motor Score; NLI: Neurological Level of Injury; Coef: coefficient; Std. Err.: standard error.

##

| **Variable** | **Coef.** | **Std. Err.** | **z** | **P>\|z\|** | **[0.025** | **0.975]** | **Effect** |
| --- | --- | --- | --- | --- | --- | --- | --- |
| **Baseline (Intercept)** | 35.2 | 17.2 | 2.054 | **0.040** | 1.6 | 68.9 | Fixed |
| **Treatment Group (*Verum*)** | -3.7 | 7.2 | -0.517 | 0.605 | -17.9 | 10.4 | Fixed |
| **Site: Barcelona** | -15.9 | 24.2 | -0.655 | 0.513 | -63.3 | 31.6 | Fixed |
| **Site: Basel** | 3.3 | 12.8 | 0.256 | 0.798 | -21.7 | 28.3 | Fixed |
| **Site: Heidelberg** | -1.3 | 15.9 | -0.082 | 0.935 | -32.4 | 29.8 | Fixed |
| **Site: Hessisch Lichtenau** | 1.4 | 14.9 | 0.096 | 0.924 | -27.7 | 30.6 | Fixed |
| **Site: Murnau** | 2.2 | 15.6 | 0.139 | 0.889 | -28.4 | 32.8 | Fixed |
| **Site: Nottwil** | -1.5 | 8.2 | -0.181 | 0.857 | -17.6 | 14.7 | Fixed |
| **Site: Prague** | -53.0 | 21.6 | -2.453 | **0.014** | -95.4 | -10.7 | Fixed |
| **Site: Tübingen** | -37.9 | 19.2 | -1.968 | **0.049** | -75.6 | -0.2 | Fixed |
| **Site: Zurich** | 5.8 | 8.9 | 0.66 | 0.509 | -11.5 | 23.2 | Fixed |
| **NLI: C2** | 0.0 | 1.3 | 0.013 | 0.989 | -2.6 | 2.7 | Fixed |
| **NLI: C3** | 0.0 | 1.3 | 0.004 | 0.997 | -2.6 | 2.6 | Fixed |
| **NLI: C4** | 0.0 | 1.3 | 0.008 | 0.994 | -2.6 | 2.6 | Fixed |
| **NLI: C5** | 0.0 | 1.4 | 0.015 | 0.988 | -2.7 | 2.7 | Fixed |
| **NLI: C6** | 0.0 | 1.4 | 0.012 | 0.991 | -2.7 | 2.8 | Fixed |
| **NLI: C7** | 0.0 | 1.5 | 0.018 | 0.986 | -2.9 | 2.9 | Fixed |
| **NLI: no NLI** | 0.0 | 2.1 | 0.021 | 0.983 | -4.0 | 4.1 | Fixed |
| **Week since injury** | 0.9 | 0.4 | 2.584 | **0.010** | 0.2 | 1.6 | Fixed |
| **Interaction Effect:**  **Week x Treatment Group** | 0.2 | 0.5 | 0.463 | 0.644 | -0.7 | 1.1 | Fixed |
| **Age at Injury** | -0.1 | 0.2 | -0.447 | 0.655 | -0.5 | 0.3 | Fixed |
| **Sex (Male=1, Female=0)** | -21.7 | 9.6 | -2.272 | **0.023** | -40.4 | -3.0 | Fixed |
| **Number of Measurements** | -0.6 | 0.5 | -1.184 | 0.236 | -1.7 | 0.4 | Fixed |
| **First measured EE** | 0.1 | 0.0 | 4.986 | **<0.001** | 0.0 | 0.1 | Fixed |
| **Week of first measured EE** | -0.3 | 1.4 | -0.217 | 0.828 | -3.0 | 2.4 | Fixed |
| **Participant-Specific EE Variability** | 19.6 | 5.0 |  |  |  |  | Random |
| **Covariance: Intercept and Week** | -0.7 | 0.3 |  |  |  |  | Random |
| **Week-Specific EE Variability** | 0.1 | 0.0 |  |  |  |  | Random |

##

## **Model F.** Mixed linear model analyzing the relationship between SCIM and key predictors for the entire cohort with sensor data. Fixed effects include treatment group, clinical site, week since injury, age at injury, sex, NLI, and related interactions. Random effects account for participant-specific variability and changes over time in intercepts and slopes. Bayreuth was selected as the reference site because it had the largest cohort. Significances (p <0.05) are indicated in bold font. Abbreviations: SCIM: Spinal Cord Independence Measure III; NLI: Neurological Level of Injury; Coef: coefficient; Std. Err.: standard error.

##

| **Variable** | **Coef.** | **Std. Err.** | **z** | **P>\|z\|** | **[0.025** | **0.975]** | **Effect** |
| --- | --- | --- | --- | --- | --- | --- | --- |
| **Baseline (Intercept)** | 56.8 | 104.3 | 0.544 | 0.586 | -147.7 | 261.3 | Fixed |
| **Treatment Group (Verum)** | -32.3 | 46.4 | -0.696 | 0.486 | -123.1 | 58.6 | Fixed |
| **Site: Barcelona** | 70.4 | 122.2 | 0.576 | 0.565 | -169.2 | 309.9 | Fixed |
| **Site: Basel** | -78.9 | 51.8 | -1.523 | 0.128 | -180.5 | 22.6 | Fixed |
| **Site: Heidelberg** | 64.8 | 61.8 | 1.049 | 0.294 | -56.3 | 186.0 | Fixed |
| **Site: Hessisch Lichtenau** | 247.0 | 87.5 | 2.822 | **0.005** | 75.5 | 418.5 | Fixed |
| **Site: Murnau** | 37.3 | 63.1 | 0.592 | 0.554 | -86.3 | 161.0 | Fixed |
| **Site: Nottwil** | 163.2 | 50.0 | 3.262 | **0.001** | 65.1 | 261.3 | Fixed |
| **Site: Prague** | 86.4 | 91.6 | 0.943 | 0.346 | -93.2 | 266.0 | Fixed |
| **Site: Tübingen** | 125.1 | 106.6 | 1.174 | 0.240 | -83.8 | 334.0 | Fixed |
| **Site: Zurich** | 48.3 | 55.7 | 0.867 | 0.386 | -60.9 | 157.5 | Fixed |
| **NLI: C2** | -1.1 | 34.5 | -0.032 | 0.974 | -68.7 | 66.5 | Fixed |
| **NLI: C3** | -2.7 | 34.8 | -0.076 | 0.939 | -70.9 | 65.6 | Fixed |
| **NLI: C4** | -2.8 | 35.1 | -0.08 | 0.936 | -71.6 | 66.0 | Fixed |
| **NLI: C5** | -3.1 | 35.8 | -0.085 | 0.932 | -73.3 | 67.2 | Fixed |
| **NLI: C6** | -0.2 | 36.7 | -0.006 | 0.995 | -72.2 | 71.7 | Fixed |
| **NLI: C7** | -6.3 | 50.3 | -0.126 | 0.900 | -105.0 | 92.3 | Fixed |
| **NLI: no NLI** | 0.4 | 50.5 | 0.009 | 0.993 | -98.6 | 99.5 | Fixed |
| **Week since injury** | 15.0 | 6.2 | 2.422 | **0.015** | 2.9 | 27.2 | Fixed |
| **Interaction Effect:**  **Week x Treatment Group** | 16.2 | 8.1 | 2.004 | **0.045** | 0.4 | 32.1 | Fixed |
| **Age at Injury** | -1.2 | 1.1 | -1.076 | 0.282 | -3.3 | 1.0 | Fixed |
| **Sex (Male=1, Female=0)** | 123.0 | 48.4 | 2.539 | **0.011** | 28.1 | 218.0 | Fixed |
| **Number of Measurements** | -4.3 | 3.1 | -1.394 | 0.163 | -10.4 | 1.7 | Fixed |
| **First measured EE** | 0.9 | 0.1 | 13.731 | **0.001** | 0.8 | 1.1 | Fixed |
| **Week of first measured EE** | -28.6 | 10.1 | -2.824 | **0.005** | -48.4 | -8.7 | Fixed |
| **Participant-Specific EE Variability** | 0.9 | 0.3 |  |  |  |  | Random |
| **Covariance: Intercept and Week** | -0.1 | 0.0 |  |  |  |  | Random |
| **Week-Specific EE Variability** | 0.0 | 0.0 |  |  |  |  | Random |

##

## **Model G.** Mixed linear model analyzing the relationship between weekly EE and key predictors for incomplete injuries. Fixed effects include treatment group, clinical site, week since injury, age at injury, sex, NLI, and related interactions. Random effects account for participant-specific variability and changes over time in intercepts and slopes. Bayreuth was selected as the reference site because it had the largest cohort. Significances (p <0.05) are indicated in bold font. Abbreviations: EE: energy expenditure; NLI: Neurological Level of Injury; Coef: coefficient; Std. Err.: standard error.

| **Variable** | **Coef.** | **Std. Err.** | **z** | **P>\|z\|** | **[0.025** | **0.975]** | **Effect** |
| --- | --- | --- | --- | --- | --- | --- | --- |
| **Baseline (Intercept)** | 31.2 | 10.4 | 2.999 | **0.003** | 10.8 | 51.7 | Fixed |
| **Treatment Group (Verum)** | -1.2 | 3.8 | -0.313 | 0.754 | -8.6 | 6.2 | Fixed |
| **Site: Basel** | -8.6 | 5.9 | -1.451 | 0.147 | -20.3 | 3.0 | Fixed |
| **Site: Heidelberg** | -7.9 | 7.4 | -1.055 | 0.291 | -22.4 | 6.7 | Fixed |
| **Site: Hessisch Lichtenau** | 3.5 | 10.1 | 0.344 | 0.731 | -16.4 | 23.3 | Fixed |
| **Site: Murnau** | 1.3 | 8.0 | 0.168 | 0.866 | -14.3 | 17.0 | Fixed |
| **Site: Nottwil** | -2.6 | 5.6 | -0.465 | 0.642 | -13.6 | 8.4 | Fixed |
| **Site: Prague** | 10.6 | 10.7 | 0.99 | 0.322 | -10.3 | 31.4 | Fixed |
| **Site: Tübingen** | -7.7 | 11.7 | -0.655 | 0.512 | -30.7 | 15.3 | Fixed |
| **Site: Zurich** | -8.0 | 6.6 | -1.205 | 0.228 | -21.0 | 5.0 | Fixed |
| **NLI: C2** | 0.0 | 0.6 | 0.018 | 0.986 | -1.1 | 1.2 | Fixed |
| **NLI: C3** | 0.0 | 0.6 | 0.023 | 0.982 | -1.2 | 1.2 | Fixed |
| **NLI: C4** | 0.0 | 0.6 | 0.027 | 0.979 | -1.2 | 1.2 | Fixed |
| **NLI: C5** | 0.0 | 0.6 | 0.03 | 0.976 | -1.2 | 1.2 | Fixed |
| **NLI: C6** | 0.0 | 0.6 | 0.035 | 0.972 | -1.2 | 1.3 | Fixed |
| **NLI: C7** | 0.0 | 0.9 | 0.021 | 0.983 | -1.7 | 1.7 | Fixed |
| **NLI: no NLI** | 0.0 | 0.8 | 0.025 | 0.980 | -1.6 | 1.7 | Fixed |
| **Week since injury** | 0.4 | 0.1 | 3.001 | **0.003** | 0.1 | 0.7 | Fixed |
| **Interaction Effect:**  **Week x Treatment Group** | 0.1 | 0.2 | 0.587 | 0.557 | -0.3 | 0.5 | Fixed |
| **Age at Injury** | -0.1 | 0.1 | -0.874 | 0.382 | -0.4 | 0.1 | Fixed |
| **Sex (Male=1, Female=0)** | 4.1 | 5.6 | 0.734 | 0.463 | -6.8 | 15.0 | Fixed |
| **Number of Measurements** | -0.3 | 0.4 | -0.857 | 0.391 | -1.0 | 0.4 | Fixed |
| **First measured EE** | 0.0 | 0.0 | 3.508 | **<0.001** | 0.0 | 0.0 | Fixed |
| **Week of first measured EE** | -1.1 | 1.4 | -0.788 | 0.431 | -3.9 | 1.7 | Fixed |
| **Participant-Specific EE Variability** | 24.1 | 7.8 |  |  |  |  | Fixed |
| **Covariance: Intercept and Week** | 0.0 | 0.3 |  |  |  |  | Random |
| **Week-Specific EE Variability** | 0.1 | 0.0 |  |  |  |  | Random |

##

## **Model H.** Mixed linear model analyzing the relationship between UEMS and key predictors for incomplete injuries. Fixed effects include treatment group, clinical site, week since injury, age at injury, sex, NLI, and related interactions. Random effects account for participant-specific variability and changes over time in intercepts and slopes. Bayreuth was selected as the reference site because it had the largest cohort. Significances (p <0.05) are indicated in bold font. Abbreviations: UEMS: Upper Extremity Motor Score; NLI: Neurological Level of Injury; Coef: coefficient; Std. Err.: standard error.

| **Variable** | **Coef.** | **Std. Err.** | **z** | **P>\|z\|** | **[0.025** | **0.975]** | **Effect** |
| --- | --- | --- | --- | --- | --- | --- | --- |
| **Baseline (Intercept)** | 39.2 | 24.3 | 1.617 | 0.106 | -8.3 | 86.8 | Fixed |
| **Treatment Group (Verum)** | 6.3 | 10.7 | 0.589 | 0.556 | -14.7 | 27.4 | Fixed |
| **Site: Basel** | -4.4 | 15.8 | -0.275 | 0.784 | -35.4 | 26.7 | Fixed |
| **Site: Heidelberg** | 4.2 | 20.5 | 0.204 | 0.838 | -35.9 | 44.3 | Fixed |
| **Site: Hessisch Lichtenau** | 8.5 | 26.4 | 0.32 | 0.749 | -43.2 | 60.1 | Fixed |
| **Site: Murnau** | 14.7 | 22.4 | 0.657 | 0.511 | -29.2 | 58.7 | Fixed |
| **Site: Nottwil** | 11.0 | 16.1 | 0.685 | 0.493 | -20.5 | 42.6 | Fixed |
| **Site: Tübingen** | -34.8 | 27.8 | -1.251 | 0.211 | -89.3 | 19.7 | Fixed |
| **Site: Zurich** | 12.2 | 15.9 | 0.769 | 0.442 | -18.9 | 43.3 | Fixed |
| **NLI: C2** | 0.1 | 2.4 | 0.039 | 0.969 | -4.5 | 4.7 | Fixed |
| **NLI: C3** | 0.1 | 2.4 | 0.036 | 0.971 | -4.6 | 4.8 | Fixed |
| **NLI: C4** | 0.1 | 2.4 | 0.043 | 0.966 | -4.6 | 4.8 | Fixed |
| **NLI: C5** | 0.1 | 2.4 | 0.039 | 0.969 | -4.7 | 4.9 | Fixed |
| **NLI: C6** | 0.1 | 2.5 | 0.034 | 0.973 | -4.8 | 5.0 | Fixed |
| **NLI: C7** | 0.1 | 3.2 | 0.024 | 0.981 | -6.2 | 6.3 | Fixed |
| **NLI: no NLI** | 0.1 | 3.1 | 0.036 | 0.971 | -6.0 | 6.3 |  |
| **Week since injury** | 1.3 | 0.6 | 2.17 | **0.030** | 0.1 | 2.4 | Fixed |
| **Interaction Effect:**  **Week x Treatment Group** | 0.0 | 0.8 | 0.015 | 0.988 | -1.5 | 1.5 | Fixed |
| **Age at Injury** | -0.1 | 0.3 | -0.222 | 0.824 | -0.7 | 0.5 | Fixed |
| **Sex (Male=1, Female=0)** | -28.0 | 14.7 | -1.911 | 0.056 | -56.8 | 0.7 | Fixed |
| **Number of Measurements** | -1.2 | 0.9 | -1.398 | 0.162 | -3.0 | 0.5 | Fixed |
| **First measured EE** | 0.1 | 0.0 | 4.303 | **<0.001** | 0.0 | 0.1 | Fixed |
| **Week of first measured EE** | -1.3 | 3.7 | -0.341 | 0.733 | -8.5 | 6.0 | Fixed |
| **Participant-Specific EE Variability** | 14.1 | 6.1 |  |  |  |  | Random |
| **Covariance: Intercept and Week** | -0.6 | 0.4 |  |  |  |  | Random |
| **Week-Specific EE Variability** | 0.1 | 0.0 |  |  |  |  | Random |

##

## **Model I.** Mixed linear model analyzing the relationship between SCIM and key predictors or incomplete injuries. Fixed effects include treatment group, clinical site, week since injury, age at injury, sex, NLI, and related interactions. Random effects account for participant-specific variability and changes over time in intercepts and slopes. Bayreuth was selected as the reference site because it had the largest cohort. Significances (p <0.05) are indicated in bold font. Abbreviations: SCIM: Spinal Cord Independence Measure III; NLI: Neurological Level of Injury; Coef: coefficient; Std. Err.: standard error.

| **Variable** | **Coef.** | **Std. Err.** | **z** | **P>\|z\|** | **[0.025** | **0.975]** | **Effect** |
| --- | --- | --- | --- | --- | --- | --- | --- |
| **Baseline (Intercept)** | 59.5 | 191.7 | 0.31 | 0.756 | -316.2 | 435.2 | Fixed |
| **Treatment Group (*Verum*)** | -90.5 | 54.1 | -1.673 | 0.094 | -196.5 | 15.5 | Fixed |
| **Site: Barcelona** | 86.8 | 137.1 | 0.634 | 0.526 | -181.8 | 355.5 | Fixed |
| **Site: Hessisch Lichtenau** | 61.4 | 127.2 | 0.483 | 0.629 | -187.9 | 310.7 | Fixed |
| **Site: Murnau** | -42.3 | 118.0 | -0.358 | 0.720 | -273.6 | 189.1 | Fixed |
| **Site: Nottwil** | 14.8 | 71.2 | 0.209 | 0.835 | -124.7 | 154.4 | Fixed |
| **Site: Prague** | -115.2 | 155.4 | -0.741 | 0.458 | -419.9 | 189.4 | Fixed |
| **Site: Tübingen** | 111.2 | 192.0 | 0.579 | 0.562 | -265.0 | 487.4 | Fixed |
| **Site: Zurich** | 6.9 | 71.5 | 0.096 | 0.923 | -133.3 | 147.1 | Fixed |
| **NLI: C2** | 0.4 | 38.6 | 0.01 | 0.992 | -75.3 | 76.1 | Fixed |
| **NLI: C3** | 0.4 | 34.3 | 0.013 | 0.990 | -66.8 | 67.7 | Fixed |
| **NLI: C4** | 1.4 | 35.8 | 0.038 | 0.970 | -68.8 | 71.6 | Fixed |
| **NLI: C5** | 3.2 | 37.5 | 0.084 | 0.933 | -70.4 | 76.7 | Fixed |
| **NLI: C6** | 1.9 | 37.8 | 0.051 | 0.959 | -72.2 | 76.1 | Fixed |
| **NLI: C7** | 2.7 | 39.4 | 0.069 | 0.945 | -74.4 | 79.9 | Fixed |
| **Week since injury** | 7.6 | 2.5 | 3.01 | **0.003** | 2.7 | 12.5 | Fixed |
| **Interaction Effect:**  **Week x Treatment Group** | 6.6 | 3.3 | 2.015 | **0.044** | 0.2 | 13.0 | Fixed |
| **Age at Injury** | -2.1 | 2.1 | -0.967 | 0.333 | -6.3 | 2.1 | Fixed |
| **Sex (Male=1, Female=0)** | -18.1 | 88.5 | -0.204 | 0.838 | -191.7 | 155.4 | Fixed |
| **Number of Measurements** | 8.5 | 5.1 | 1.677 | 0.093 | -1.4 | 18.5 | Fixed |
| **First measured EE** | 0.9 | 0.2 | 3.918 | **<0.001** | 0.4 | 1.3 | Fixed |
| **Week of first measured EE** | -14.0 | 10.4 | -1.351 | 0.177 | -34.4 | 6.3 | Fixed |
| **Participant-Specific EE Variability** | 0.7 | 0.3 |  |  |  |  | Random |
| **Covariance: Intercept and Week** | 0.0 | 0.0 |  |  |  |  | Random |
| **Week-Specific EE Variability** | 0.0 | 0.0 |  |  |  |  | Random |

##

## **Model J.** Mixed linear model analyzing the relationship between weekly EE and key predictors for complete injuries. Fixed effects include treatment group, clinical site, week since injury, age at injury, sex, NLI, and related interactions. Random effects account for participant-specific variability and changes over time in intercepts and slopes. Bayreuth was selected as the reference site because it had the largest cohort. Significances (p <0.05) are indicated in bold font. Abbreviations: EE: energy expenditure; NLI: Neurological Level of Injury; Coef: coefficient; Std. Err.: standard error.

| **Variable** | **Coef.** | **Std. Err.** | **z** | **P>\|z\|** | **[0.025** | **0.975]** | **Effect** |
| --- | --- | --- | --- | --- | --- | --- | --- |
| **Baseline (Intercept)** | -7.9 | 14.9 | -0.53 | 0.596 | -37.2 | 21.4 | Fixed |
| **Treatment Group (*Verum*)** | -3.6 | 4.2 | -0.855 | 0.393 | -11.7 | 4.6 | Fixed |
| **Site: Barcelona** | 20.2 | 8.8 | 2.292 | **0.022** | 2.9 | 37.4 | Fixed |
| **Site: Hessisch Lichtenau** | 9.2 | 10.6 | 0.866 | 0.387 | -11.6 | 30.1 | Fixed |
| **Site: Murnau** | 9.0 | 8.4 | 1.076 | 0.282 | -7.4 | 25.4 | Fixed |
| **Site: Nottwil** | 2.7 | 5.8 | 0.462 | 0.644 | -8.7 | 14.0 |  |
| **Site: Prague** | -11.4 | 17.5 | -0.65 | 0.516 | -45.6 | 22.9 | Fixed |
| **Site: Tübingen** | 1.9 | 17.7 | 0.105 | 0.916 | -32.8 | 36.5 | Fixed |
| **Site: Zurich** | 9.3 | 5.3 | 1.766 | 0.077 | -1.0 | 19.7 | Fixed |
| **NLI: C2** | 0.0 | 0.6 | 0.014 | 0.989 | -1.2 | 1.2 | Fixed |
| **NLI: C3** | 0.0 | 0.6 | 0.012 | 0.990 | -1.1 | 1.1 | Fixed |
| **NLI: C4** | 0.0 | 0.6 | 0.025 | 0.980 | -1.1 | 1.2 | Fixed |
| **NLI: C5** | 0.0 | 0.6 | 0.035 | 0.972 | -1.2 | 1.2 | Fixed |
| **NLI: C6** | 0.0 | 0.6 | 0.028 | 0.978 | -1.2 | 1.2 | Fixed |
| **NLI: C7** | 0.0 | 0.6 | 0.033 | 0.973 | -1.2 | 1.3 | Fixed |
| **Week since injury** | 0.2 | 0.1 | 3.482 | **<0.001** | 0.1 | 0.4 | Fixed |
| **Interaction Effect:**  **Week x Treatment Group** | 0.0 | 0.1 | -0.158 | 0.874 | -0.2 | 0.2 | Fixed |
| **Age at Injury** | -0.1 | 0.2 | -0.783 | 0.433 | -0.4 | 0.2 | Fixed |
| **Sex (Male=1, Female=0)** | -8.7 | 11.2 | -0.778 | 0.436 | -30.6 | 13.2 | Fixed |
| **Number of Measurements** | 1.4 | 0.5 | 2.962 | **0.003** | 0.5 | 2.3 | Fixed |
| **First measured EE** | 0.1 | 0.0 | 2.843 | **0.004** | 0.0 | 0.1 | Fixed |
| **Week of first measured EE** | 0.1 | 0.7 | 0.153 | 0.878 | -1.3 | 1.5 | Fixed |
| **Participant-Specific EE Variability** | 16.5 | 7.5 |  |  |  |  | Random |
| **Covariance: Intercept and Week** | -0.2 | 0.2 |  |  |  |  | Random |
| **Week-Specific EE Variability** | 0.0 | 0.0 |  |  |  |  | Random |

## **Model K.** Mixed linear model analyzing the relationship between UEMS and key predictors for complete injuries. Fixed effects include treatment group, clinical site, week since injury, age at injury, sex, NLI, and related interactions. Random effects account for participant-specific variability and changes over time in intercepts and slopes. Bayreuth was selected as the reference site because it had the largest cohort. Significances (p <0.05) are indicated in bold font. Abbreviations: UEMS: Upper Extremity Motor Score; NLI: Neurological Level of Injury; Coef: coefficient; Std. Err.: standard error.

| **Variable** | **Coef.** | **Std. Err.** | **z** | **P>\|z\|** | **[0.025** | **0.975]** | **Effect** |
| --- | --- | --- | --- | --- | --- | --- | --- |
| **Baseline (Intercept)** | 7.6 | 23.0 | 0.332 | 0.740 | -37.5 | 52.7 | Fixed |
| **Treatment Group (Verum)** | -9.7 | 7.0 | -1.386 | 0.166 | -23.3 | 4.0 | Fixed |
| **Site: Basel** | 10.0 | 12.8 | 0.781 | 0.435 | -15.1 | 35.1 | Fixed |
| **Site: Heidelberg** | 10.6 | 15.3 | 0.692 | 0.489 | -19.4 | 40.7 | Fixed |
| **Site: Hessisch Lichtenau** | 7.7 | 11.9 | 0.642 | 0.521 | -15.7 | 31.1 | Fixed |
| **Site: Murnau** | 2.6 | 8.1 | 0.318 | 0.751 | -13.3 | 18.5 | Fixed |
| **Site: Nottwil** | -12.2 | 18.7 | -0.654 | 0.513 | -48.9 | 24.5 | Fixed |
| **Site: Tübingen** | -14.5 | 20.3 | -0.715 | 0.475 | -54.3 | 25.3 | Fixed |
| **Site: Zurich** | 6.0 | 8.6 | 0.7 | 0.484 | -10.8 | 22.8 | Fixed |
| **NLI: C2** | 0.0 | 1.3 | 0.003 | 0.997 | -2.6 | 2.6 | Fixed |
| **NLI: C3** | 0.0 | 1.2 | 0.006 | 0.995 | -2.4 | 2.4 | Fixed |
| **NLI: C4** | 0.0 | 1.3 | 0.015 | 0.988 | -2.5 | 2.5 | Fixed |
| **NLI: C5** | 0.0 | 1.3 | 0.036 | 0.971 | -2.5 | 2.6 | Fixed |
| **NLI: C6** | 0.0 | 1.3 | 0.025 | 0.980 | -2.5 | 2.6 | Fixed |
| **NLI: C7** | 0.0 | 1.4 | 0.036 | 0.971 | -2.6 | 2.7 | Fixed |
| **Week since injury** | 0.5 | 0.2 | 2.645 | **0.008** | 0.1 | 0.8 | Fixed |
| **Interaction Effect:**  **Week x Treatment Group** | 0.2 | 0.2 | 0.756 | 0.450 | -0.3 | 0.7 | Fixed |
| **Age at Injury** | -0.3 | 0.3 | -1.248 | 0.212 | -0.8 | 0.2 | Fixed |
| **Sex (Male=1, Female=0)** | -7.9 | 10.6 | -0.745 | 0.456 | -28.6 | 12.8 | Fixed |
| **Number of Measurements** | 0.8 | 0.8 | 1.094 | 0.274 | -0.7 | 2.3 | Fixed |
| **First measured EE** | 0.1 | 0.0 | 1.864 | 0.062 | 0.0 | 0.1 | Fixed |
| **Week of first measured EE** | 0.2 | 1.1 | 0.227 | 0.820 | -1.8 | 2.3 | Fixed |
| **Participant-Specific EE Variability** | 15.5 | 6.8 |  |  |  |  | Random |
| **Covariance: Intercept and Week** | -0.4 | 0.2 |  |  |  |  | Random |
| **Week-Specific EE Variability** | 0.0 | 0.0 |  |  |  |  | Random |

##

## **Model L.** Mixed linear model analyzing the relationship between SCIM and key predictors or complete injuries. Fixed effects include treatment group, clinical site, week since injury, age at injury, sex, NLI, and related interactions. Random effects account for participant-specific variability and changes over time in intercepts and slopes. Bayreuth was selected as the reference site because it had the largest cohort. Significances (p <0.05) are indicated in bold font. Abbreviations: SCIM: Spinal Cord Independence Measure III; NLI: Neurological Level of Injury; Coef: coefficient; Std. Err.: standard error.
